# Reproductive Isolation of Hybrid Populations Driven by Genetic Incompatibilities

**DOI:** 10.1371/journal.pgen.1005041

**Published:** 2015-03-13

**Authors:** Molly Schumer, Rongfeng Cui, Gil G. Rosenthal, Peter Andolfatto

**Affiliations:** 1 Department of Ecology and Evolutionary Biology, Princeton University, Princeton, New Jersey, United States of America; 2 Department of Biology, Texas A&M University, College Station, Texas, United States of America; 3 Centro de Investigaciones Científicas de las Huastecas “Aguazarca”, Calnali, Hidalgo, Mexico; 4 Max Planck Institute for the Biology of Ageing, Cologne, Germany; 5 Lewis-Sigler Institute for Integrative Genomics, Princeton University, Princeton, New Jersey, United States of America; University of Wisconsin—Madison, UNITED STATES

## Abstract

Despite its role in homogenizing populations, hybridization has also been proposed as a means to generate new species. The conceptual basis for this idea is that hybridization can result in novel phenotypes through recombination between the parental genomes, allowing a hybrid population to occupy ecological niches unavailable to parental species. Here we present an alternative model of the evolution of reproductive isolation in hybrid populations that occurs as a simple consequence of selection against genetic incompatibilities. Unlike previous models of hybrid speciation, our model does not incorporate inbreeding, or assume that hybrids have an ecological or reproductive fitness advantage relative to parental populations. We show that reproductive isolation between hybrids and parental species can evolve frequently and rapidly under this model, even in the presence of substantial ongoing immigration from parental species and strong selection against hybrids. An interesting prediction of our model is that replicate hybrid populations formed from the same pair of parental species can evolve reproductive isolation from each other. This non-adaptive process can therefore generate patterns of species diversity and relatedness that resemble an adaptive radiation. Intriguingly, several known hybrid species exhibit patterns of reproductive isolation consistent with the predictions of our model.

## Author Summary

Understanding the origin of species is one of the central challenges in evolutionary biology. It has been suggested that hybridization could generate new species because hybrids can display novel combinations of traits that induce reproductive isolation from their parental species (called “hybrid speciation”). Existing models predict that this should only occur in special cases, and indeed there have been only few well-supported examples. We describe a new model of hybrid reproductive isolation that results from selection against genetic incompatibilities in hybrids, which are predicted to be common. Simulations reveal that hybrid populations rapidly and frequently become isolated from parental species by fixing combinations of genes that hinder successful reproduction with parental species. We propose that this process could be an important mechanism for the formation of new species.

## Introduction

The evolutionary significance of hybridization has been a hotly debated topic for decades [1]. Homoploid hybrid speciation, speciation that occurs as a result of hybridization without a ploidy change [2, 3], is generally thought to be an exceptionally rare outcome of hybridization, and there are indeed only a handful of well-supported cases of this phenomenon [4]. Though it is not uncommon for species’ genomes to exhibit evidence of past hybridization, hybrids are often thought to be weakly isolated from parental species, though few studies have explicitly investigated this.

Empirical research on homoploid hybrid speciation over the last decade has primarily focused on the role of hybrid phenotypes in establishing reproductive isolation between hybrids and parental species [5–9]. Hybrids can have recombinant or transgressive traits that differentiate them from parental species. In some cases, these traits can allow hybrids to occupy new niches. For example, in *Rhagoletis* fruit flies, hybrid lineages have novel host preferences, potentially contributing to reproductive isolation between hybrids from parental species [10, 11]. Similarly, if hybrid lineages have novel mate preferences, this can isolate hybrids from parental species via assortative mating, a mechanism which has been implicated in hybrid speciation in *Heliconius* butterflies ([8], and see [12] for a model of this process). This work has lead to the idea that novel hybrid phenotypes are key to hybrid speciation [13].

Despite several well-documented examples [6, 8], it has been difficult to determine the evolutionary importance of hybrid speciation, in part because few theoretical models have been developed. The existing models of hybrid speciation simulate either positive selection on certain hybrid genotypes or inbreeding [9, 12, 14]. In one model [14, 15], novel combinations of underdominant parental inversions can fix in hybrid populations, particularly if the novel inversion combination is under positive selection or if rates of inbreeding (selfing) are high (see Discussion). Though there is evidence that this process combined with ecological factors was involved in the formation of hybrid *Helianthus* sunflower species [5, 6, 16], the basis for invoking positive selection on recombinant inversion genotypes is unclear. Later versions of this model incorporated ecological differentiation between hybrid and parental species and showed that hybrid speciation occurred frequently if hybrids had higher fitness than parental species in an unoccupied niche [9, 17]. Though hybridization often generates novel traits [18–20] it is difficult to evaluate the likelihood that these traits will be more fit than parental types (ecologically or intrinsically), making it difficult to predict the importance of hybridization in generating new species by positive selection on hybrid genotypes.

The genetic incompatibility of hybrids constitutes a key component of reproductive isolation between many species, and is the basis for the biological species concept. While previous models of hybrid speciation incorporated inversions [21], here we investigate the potential role of negative epistatic interactions, another important genetic mechanism of speciation. The first genetic model of speciation, described by Bateson, Dobzhansky and Muller (the BDM incompatibility model, S1 Fig., [22–24]), predicts that mutations at two genetic loci differentially accumulating along two lineages can negatively interact in their hybrids. Empirical research has shown that these types of negative epistatic interactions are remarkably common [25–29]; reviewed in [24, 30, 31].

Though the theory of genetic incompatibilities was originally formulated in the context of allopatrically diverging species, more recent research has investigated dynamics of these incompatibilities in the context of hybrid zones. Under the simplest BDM scenario, derived genotypes are presumed to be neutral, meaning that they have the same fitness as ancestral genotypes. When there is gene flow between species, neutral BDMs are predicted to fix for genotype combinations that are compatible with either parental species [32], rendering them ineffective barriers to gene flow [33, 34]. However, incompatibilities may also frequently arise due to adaptive evolution or coevolution of pairs of loci along lineages (S1 and S2 Figs [24, 32, 35–37]). Such incompatibilities are more effective barriers to gene flow than neutral BDM incompatibilities ([38], see also [39]).

**Fig 1:**
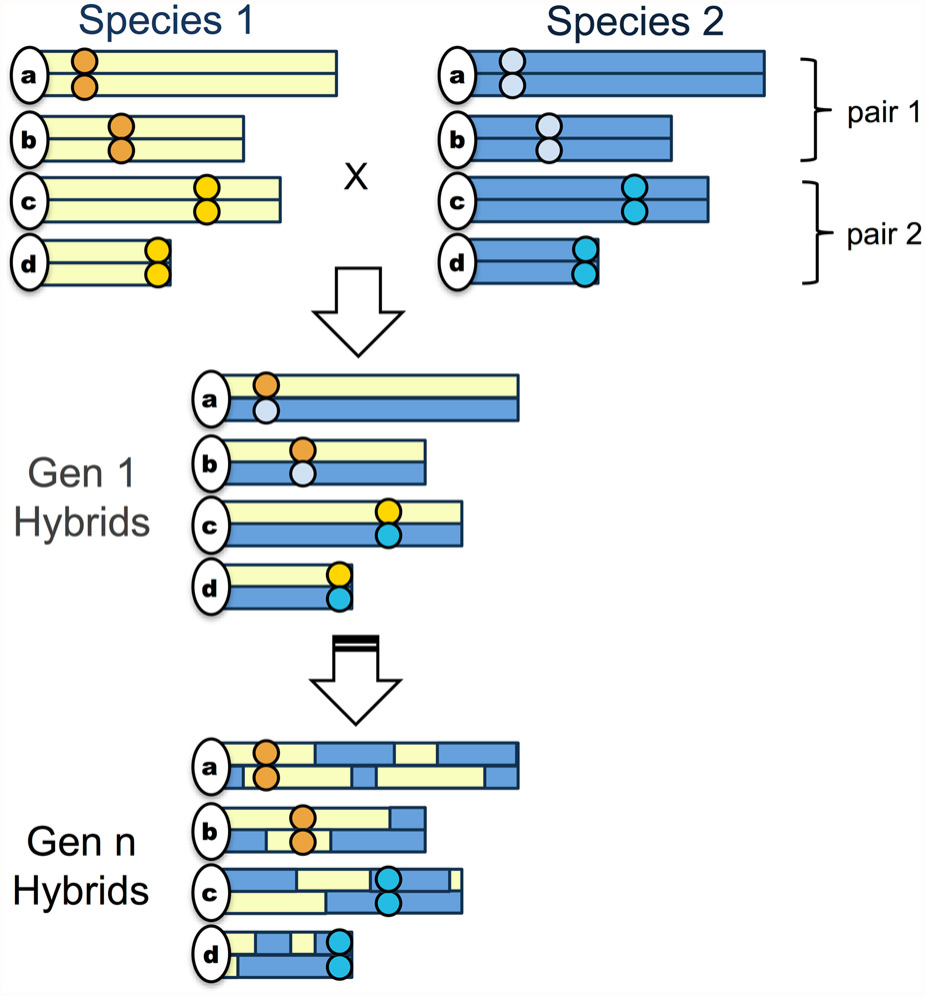
Schematic of the simplest “hybrid speciation by genetic incompatibility” scenario. The simplest model hybrid reproductive isolation evolves in a hybrid swarm (S3 Fig.) via fixation of parental genetic incompatibility pairs in opposite directions. Circles indicate the location of incompatibility pairs on chromosomes; yellow shading indicates regions derived from species 1 while blue shading indicates regions derived from species 2. In the first generation, assuming random mating, 50% of individuals will be F_1_ hybrids if both species contribute equally to the hybrid population. In subsequent generations, recombination will break up ancestry blocks and selection will drive the fixation of parental genotypes at incompatibility loci. In some proportion of cases, incompatibility pairs will fix for opposite parental species genotypes.

In its initial description, the BDM model envisioned incompatibilities that cause complete hybrid inviability or sterility, but many negative epistatic interactions in interspecific crosses have more moderate effects on fitness (e.g. [40–42]), allowing hybrid populations to persist. With few exceptions, previous work on genetic incompatibilities has focused on their role in maintaining reproductive isolation between parental species. As a result, hybrid populations have primarily been modeled as tension zones, but incompatibilities may also have interesting dynamics within isolated hybrid populations (i.e. hybrid swarms). Here we present a new model in which reproductive isolation between hybrid and parental populations emerges as a consequence of selection against incompatibilities in a hybrid swarm. Selection on a single adaptive or coevolving incompatibility pair can result in the fixation of genotype combinations that contribute to isolation between the hybrid population and one or the other parental species. Here, we show that in the presence of multiple pairs of such incompatibilities, this process can result in the rapid evolution of reproductive isolation of hybrid populations from both parental species (Fig. 1).

Two features of this model make it particularly plausible biologically. First, as noted above, negative epistatic interactions are common, providing ample raw material for our model. Second, hybrid populations in which hybrids are abundant are common in nature (e.g. [43–45]). Though ecological and sexual selection are important factors in the few well-documented cases of hybrid speciation [6, 8], our results suggest that hybrids can evolve reproductive isolation as a result of selection against genetic incompatibilities alone.

## Results

### Modeling selection against hybrid incompatibilities

In the simplest model of a hybrid population, an equal mixture of individuals from both parental species form a new isolated population and mate randomly with respect to genotype (Figs. 1 and S3), such that the first mating event generates 50% F_1_ hybrids and 25% each parental species. Using theory of two-locus selection [46, 47], hereafter the “deterministic two-locus model”, one can model the effect of selection at two polymorphic loci on gamete frequencies of a diploid sexual population (see Methods and S1 Text). This model describes the dynamics of two loci subject to any arbitrary fitness matrix. Here, we focus on fitness matrices for three types of incompatibilities that may commonly arise between species (S1 and S2 Figs; [30, 35]): 1. BDM incompatibilities arising from neutral substitutions, 2. BDM incompatibilities arising from adaptive substitutions, and 3. BDM incompatibilities arising from coevolution between loci. Applying the two-locus selection model to these incompatibility types, one can see that the direction of fixation depends on the initial frequency of the parental alleles (*f*, see S3 and S4 Figs) and dominance at each locus (*h*, S4 Fig.; see also [48]).

**Fig 2:**
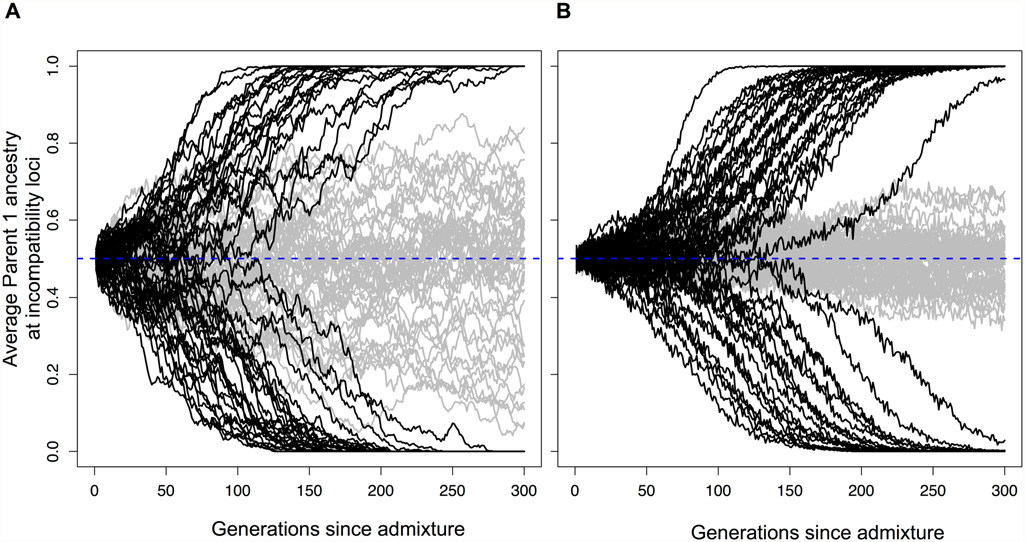
Hybrid populations rapidly fix for hybrid incompatibility locus pairs. Selection drives hybrid incompatibility loci to fixation, even when a hybrid population forms at equal admixture proportions (*f =* 0.5). Black lines show average parent 1 ancestry at a hybrid incompatibility pair (*h* = 0.5, *s* = 0.1) in 50 replicate populations of (A) *N* = 1,000 or (B) *N* = 10,000 diploid individuals. Gray lines show results for this same population size with no selection. Because of this behavior, two incompatibility pairs may fix for opposite parents, resulting in reproductive isolation of hybrids from both parents (see Fig. 1).

This purely deterministic model of selection on hybrid incompatibilities is unrealistic because even large populations experience some degree of genetic drift. We thus extended the model to include genetic drift, which can affect the speed and direction of fixation of incompatibility pairs (S5 Fig.). For neutral BDM incompatibilities (S1 Fig.), this model does not predict fixation of genotypes incompatible with either parental species (S4 Fig.). In contrast, for coevolving or adaptive BDM incompatibilities (S1 Text, S1 and S2 Figs), the two-locus finite population model predicts that at equal admixture proportions (*f* = 0.5), a single incompatibility pair has a 50% chance of fixing for either parental allele combination (Fig. 2, S2 Text, S1 Table). Interestingly, while genetic drift in small populations could accomplish the same thing (9), the process described here occurs rapidly in large populations and is driven by deterministic selection (Fig. 2). Given these dynamics, it is clear that large hybrid populations with two or more of these types of hybrid incompatibilities could, in principle, fix for one parental genotype at one incompatibility pair and the other parental genotype at the other incompatibility pair (Fig. 1). This outcome would result in reproductive isolation of the hybrid population from both parental species. With two codominant incompatibility pairs and equal admixture proportions, the probability that a hybrid population will become isolated can be predicted by a simple binomial. However the binomial prediction breaks down when there is variation in dominance, admixture proportions, or linkage between incompatibilities, and thus we explore these further by simulation.

### Simulations of an isolated hybrid population

To investigate the dynamics of multiple incompatibility pairs, we simulated a large, randomly-mating and spatially isolated hybrid population with two pairs of unlinked hybrid incompatibility loci (see Methods; S3 Fig., setting *m*_*1*_ = *m*_*2*_ = 0). The fitness scheme used is that of a coevolutionary incompatibility model (S2 Fig.), assuming that incompatibilities are codominant (i.e. *h* = 0.5), that fitness is symmetric with respect to the parental source of alleles (i.e. *w*_*ij*_ = *w*_*ji*_) and that the cumulative fitness effects of multiple incompatibility pairs is multiplicative. If hybrid populations fixed for the parent species 1 genotype at one incompatibility pair and the parent species 2 genotype at the other, we considered the hybrid population as having evolved reproductive isolation from both parental species (albeit weaker than between the two parental species).

**Fig 3:**
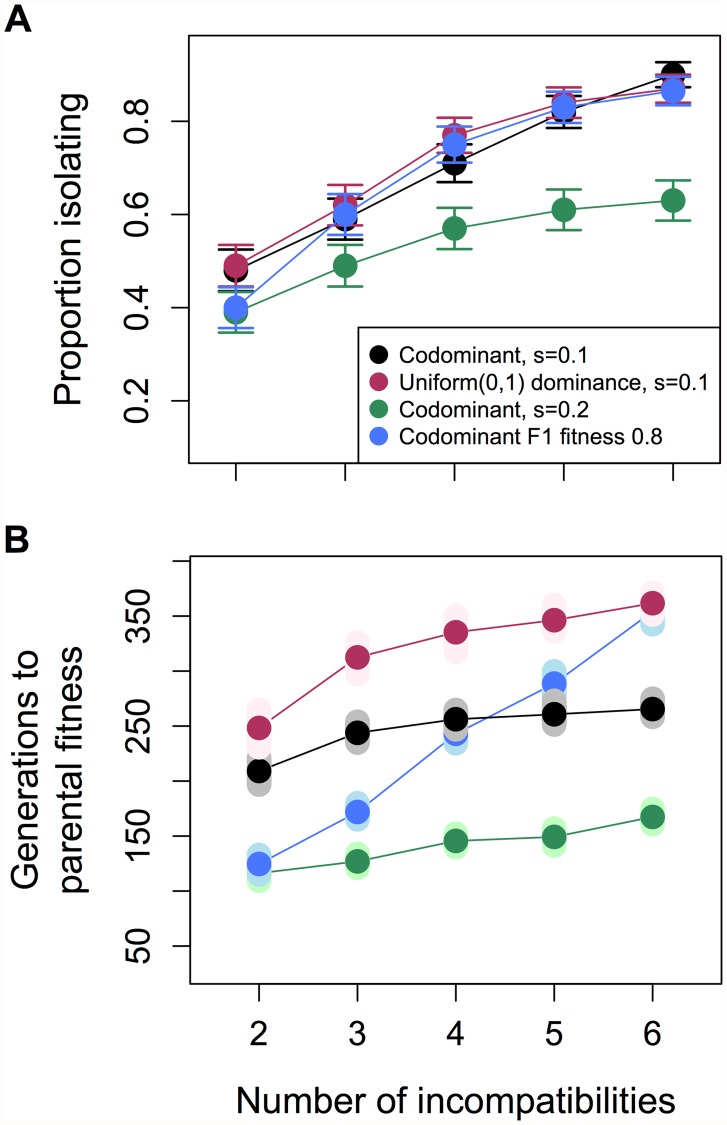
Relationship between the number of hybrid incompatibility pairs and probability of evolving isolation from both parents. With an increasing number of hybrid incompatibility pairs, reproductive isolation from both parents increases in likelihood (A) but populations require longer periods of time to reach parental fitness levels (B). In these simulations two to six hybrid incompatibility pairs distinguish the hybridizing species and hybrid populations formed at equal admixture proportions (*f* = 0.5, 1,000 diploid individuals). Simulations labeled F_1_ indicate that the selection coefficients were set such that the fitness of F_1_ hybrids between the two parental species equaled 0.8 regardless of the number of incompatibilities. Results are based on 500 replicate simulations. In (A) whiskers represent two standard errors; in (B) smears represent the means of 1,000 bootstrap samples.

While selection against hybrids will sometimes be so extreme that few hybrids will survive (or reproduce) in the population (see simulations below), selection against hybrids can also be more moderate, allowing hybrids to persist [41, 45, 49–53]. In simulations of this moderate selection scenario, reproductive isolation between hybrid and parental populations can evolve frequently and rapidly (Fig. 3). For example, for two incompatibility pairs with selection coefficients (*s*) of 0.1, 47±2% of simulated hybrid populations became isolated from both parental species within an average of ~200 generations. Exploring a range of *s* (0.1–0.5, S6 Fig., S2 Table), initial admixture proportions (*f* = 0.3–0.7, S7 Fig.), and population sizes (100–10,000 diploids, S3 Table), we conclude that, unless fitness of hybrids is low (i.e. F_1_ fitness <0.5) or ancestry of the founding population is substantially skewed (>60% one parental species), reproductive isolation evolves rapidly and with surprisingly high probability (27±2% to 43±2% of the time; on average within 75 ± 16 to 258 ± 38 generations, see S3 Text).

### The effect of dominance and asymmetry in selection intensity

In the above simulations, we assume that selection on different hybrid incompatibility interactions is symmetrical (*s*_1_ = *s*_2_, S2 Fig.), but it is unlikely that selection is truly equal on different hybrid genotype combinations. When fitness is completely asymmetrical (i.e. *s*_1_ = 0 in S1 Fig., as for neutral BDM incompatibilities), only strong genetic drift can cause the fixation of genotype pairs that are incompatible with either parental species, as selection cannot do so (see S4, S8, S9 Figs, S4 Text). This reliance on genetic drift implies that this process will be slow unless an extreme bottleneck is invoked.

In contrast, the dynamics of BDM incompatibilities resulting from adaptation within parental lineages can be quite different (S1 Fig.). Notably, while selection may also be highly asymmetric in such cases [38, 54], derived alleles have higher fitness than ancestral alleles, allowing for the fixation of genotype combinations that are incompatible with both parental species. We find that isolation evolves with similar frequency under asymmetric selection as long as selection is strong relative to drift (S3 Text, S4 Table), because even weak selection will prevent the fixation of the ancestral genotype.

Above we simulated codominant hybrid incompatibilities (*h* = 0.5), but the two-locus model (S4 Fig.) shows that patterns of fixation are different depending on the value of *h*. In particular, when *h* is zero or unity, fixation is not strongly dependent on admixture proportions (S4 Fig.). When we simulate variation in dominance among incompatibility interactions (see S3 Text, S5 Table), we find that reproductive isolation between hybrid populations and parental species evolves with comparable frequency (42–48±2% vs 47±2% under the codominant scenario).

### Increasing the number of hybrid incompatibilities

Recent empirical studies have suggested that most species are distinguished by multiple hybrid incompatibilities [30, 41, 55–59]. Theoretically, barring extinction of the hybrid population (see simulations below), increasing the number of pairs of incompatibilities should increase the probability that a hybrid population will evolve isolation from both parental species. In order to illustrate this, we simulated 3–6 unlinked hybrid incompatibility pairs (S5 Text). As expected, increasing the number of hybrid incompatibilities increases the probability that the hybrid population will be isolated from each parental species by at least one incompatibility pair (>90% with 6 incompatibility pairs, Figs. 3, S6, S5 Text).

We assume in most of our simulations that loci involved in hybrid incompatibilities are completely unlinked. As the number of incompatibilities increases, this becomes unlikely. Genetic linkage between loci involved in different epistatic interactions can reduce the frequency at which hybrid populations evolve isolation because alleles are more likely to fix for the same parental genotype (S10 Fig., S5 Text, S6 Table). Interestingly, when coevolving incompatibility loci are linked to a neutral BDM incompatibility, this does not significantly lower the frequency at which hybrid populations evolve reproductive isolation (S5 Text). Furthermore, linkage between coevolving incompatibilities and neutral BDM incompatibilities can more frequently result in fixation of neutral BDM incompatibilities for a parental genotype (16±2%), resulting in stronger isolation between hybrid and parental populations (S5 Text).

**Fig 4:**
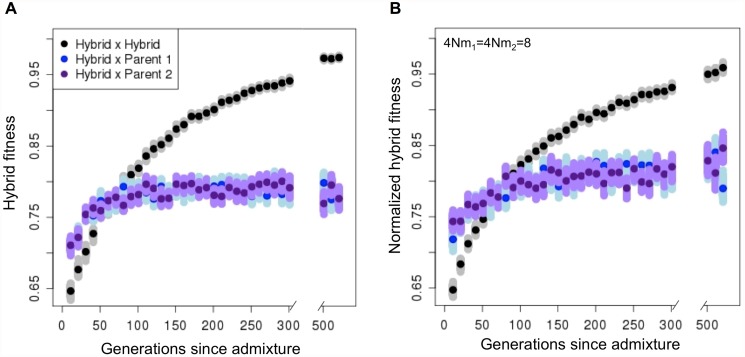
Hybrid populations rapidly develop reproductive isolation from both parental species, even in the presence of migration. Once hybrid populations diverge in ancestry at hybrid incompatibility loci from parental populations, individual hybrids have higher fitness on average when they mate with other hybrids in their population compared to either parent. (A) No migration and (B) ongoing migration (4*Nm*_1_ = 4*Nm*_2_ = 8) from parental populations. Dark points represent the mean fitness, and smears represent the means of 1,000 bootstrap samples. In B, fitness is normalized to the mean fitness of individuals in the parental populations. Simulation parameters: 100 replicates per time point, *N* = 1000, 20 hybrid incompatibility pairs, *s*_*1*_, *s*_*2*_ and *h* drawn from distributions (exponential, exponential and uniform, respectively, see details S5 Text).

The above simulations focus on simple models that show this process can occur in principle. To capture more biological realism in the number and types of incompatibilities, we simulated 20 incompatibility pairs with randomly determined genomic position and dominance, exponentially distributed selection coefficients (mean *s* = 0.05) and variation in asymmetry of selection (see above and S5 Text). In these simulations, 95% of populations developed isolation from both parental species. On average, the hybrid population first evolved isolation from both parental species after ~250 generations and was isolated from each by 7 incompatibility pairs within 1000 generations. Since incompatibility pairs with the largest fitness effects tend to fix first, hybrid populations developed considerable reproductive isolation from parental species even before all incompatibilities were fixed in the population (Figs. 4 and S11). Overall, our simulations suggest that rapid evolution of reproductive isolation of hybrid populations is likely when parental species are separated by several hybrid incompatibility pairs.

Reproductive isolation between hybrids and parental species is less likely to evolve as the fitness of hybrids decreases. For example, if we repeat the simulations above (i.e. the 20 incompatibility pairs with exponentially distributed selection coefficients), if $\overset{-}{s}$ = 0.1, the average fitness of an F_1_ hybrid between the two parental species is 0.38 and isolation evolves in only 56±2% of simulations. When $\overset{-}{s}$ = 0.2, the average fitness of hybrids is 0.1, and only 1.4±0.5% of simulated populations develop isolation and parental genotypes dominated in these populations. Thus, this process is unlikely to occur between species in which post-zygotic isolation is nearly complete.

Similarly, if parental individuals in the simulated hybrid population mate assortatively with conspecifics, reproductive isolation between hybrids and parental species is significantly less likely to evolve (S6 Text). The reasons for this are two-fold: assortative mating prevents the formation of a large hybrid population, and parentals outcompete early generation hybrids that are still segregating for parental incompatibilities.

### Simulations of hybrid populations with ongoing migration

We model a completely isolated hybrid swarm, but many hybrid populations experience gene flow with parental species. Ongoing migration may impede the evolution of reproductive isolation by preventing the fixation of genetic incompatibilities. To evaluate this, we simulated hybridization scenarios with ongoing migration (S3 and S13 Figs, 4*Nm* = 8–20). Even with substantial gene flow from parental populations, hybrid populations evolved reproductive isolation from them at high probability (i.e. 38±2% of simulations with two incompatibility pairs, *s* = 0.1 and 4*Nm* = 8; S6 Text; S7 Table).

In the above simulations, we assume that migration is symmetrical from both parental species, but asymmetrical migration may be common in hybrid zones (e.g. [60–62]). To explore how asymmetrical migration could influence our results, we varied asymmetry in migration rates (S6 Text). As expected, when migration rates were high and strongly asymmetrical (S12 Fig.), hybrid reproductive isolation from both parental species evolved infrequently. However, in less extreme cases, hybrid reproductive isolation was still observed frequently (e.g. >20% of simulations with 4*Nm*<20, S12 Fig.).

It is interesting to consider the fact that chance plays an important role in the direction that incompatibility pairs fix. As a result, one would expect that two or more independently formed hybrid populations from the same pair of parental species could evolve isolation from each other. To demonstrate this effect, we simulated two hybrid populations formed from the same pair of parental species (S14 Fig.). In the absence of migration, the two hybrid populations evolved isolation from each other frequently (50±5%, as expected given two hybrid incompatibility pairs, see S6 Text; S8 Table). Remarkably, this outcome is still observed with relatively high gene flow between the two hybrid populations (24±4% with 4*Nm* = 8 and two hybrid incompatibility pairs, S6 Text; S8 Table).

## Discussion

We describe a new model of the evolution of reproductive isolation of hybrid populations, a first step towards hybrid speciation. Unlike previous models of hybrid speciation, our model does not assume positive selection on hybrid genotypes or inbreeding, but rather deterministic selection against hybrid incompatibilities in randomly mating hybrid populations. With moderate selection (i.e. *s*≤0.2) on two or more incompatibility pairs in an allopatric hybrid population, reproductive isolation from both parental species emerges with ~50% (or higher) probability. Hybrid reproductive isolation also evolves frequently with substantial levels of ongoing migration between hybrids and parental species (4*Nm* < 20 each parent).

Another striking result of our simulations is the speed with which reproductive isolation evolves between hybrids and parental species. Depending on parameters, reproductive isolation can emerge in fewer than 100 generations with moderate selection (S3 Text). The idea that hybrid speciation can occur rapidly has been supported by experimental results [14, 63, 64] and to some extent by previous models of hybrid speciation [9, 14]. Our model suggests that simple selection on incompatibilities in hybrid populations could also lead to rapid reproductive isolation on timescales much faster than expected for allopatric speciation due to the accumulation of neutral BDM incompatibilities. Given that epistatic incompatibilities are common, our results on the probability and speed of isolation suggest that this process may frequently occur in hybrid populations.

Previous empirical work has emphasized the importance of ecological differentiation between hybrid and parental populations or positive selection on hybrid genotypes as a route to hybrid reproductive isolation [6, 8–10, 12, 63, 65]. The novel finding of our simulations is that reproductive isolation evolves readily in hybrid populations without positive selection on hybrids. However, the two are not mutually exclusive and ecological factors, which have been shown to underlie several cases of hybrid speciation [6, 8, 63], may complement selection on genetic incompatibilities to further strengthen reproductive isolation. For example, in *Helianthus*, a combination of chromosomal rearrangements and novel hybrid phenotypes are important in distinguishing hybrid and parental species [6, 66]. Like other models ([9, 14]), our model predicts that isolation between hybrids and parental species is inherently weaker than isolation between the two parental species. We propose that fixation of incompatibilities could be a crucial step in initially limiting gene flow between hybrids and parental species, allowing for the development of other isolating mechanisms. For example, theoretical work predicts that reinforcement can develop even when selection against gene flow is moderate [67–70].

Previous models of hybrid speciation have incorporated species-specific inversions that are assumed to be underdominant. Under this “underdominant inversion” model, hybrid populations can fix for novel inversion combinations, resulting in isolation between hybrid and parental species [15]. Simulation results under this model have suggested that inbreeding [14] or positive selection on hybrid genotypes [9, 14] is important for the evolution of hybrid reproductive isolation. However, past simulation efforts focused on hybrids in a tension zone, either with no spatial isolation from parental species [14] or with high migration rates from parental species [17]. To investigate the dynamics of the underdominant inversion model in situations where migration is more restricted, we simulate the underdominant inversion model in an isolated hybrid swarm scenario that is similar to our epistatic incompatibility model (S7 Text). Interestingly, we find that isolation evolves frequently under this model even without positive selection (~40% of simulations, see S7 Text). These results show that, in hybrid-dominated populations, the inversion model has similar behavior to our model of selection against negative epistatic interactions (S7 Text). Which mechanism of isolation is more prevalent in hybrid populations will depend on the frequency of hybrid incompatibilities of each type. Empirical evidence suggests that while underdominance can be a common isolating mechanism in plants (reviewed in [21]), negative epistatic interactions may be a more common mechanism of reduced hybrid fitness in animals [24].

It is important to note several factors that may influence how common our epistatic interactions model of hybrid speciation will be in natural populations. First, our model assumes that hybrids are abundant in a population and, while this appears to be reasonably common (see S6 Text; S9 Table), this is clearly not a feature of all hybrid zones. We also note that our model only represents fitness in terms of genetic incompatibilities and that hybrid populations can have lower fitness as a result of ecological or sexual selection. For example, in our simulations, we assumed random mating between hybrids and parentals. But when parental species exert negative sexual selection against hybrids, hybrid populations are significantly more likely to be outcompeted by parentals (S10 Table). There is substantial variation in the mating preferences of parentals for hybrids [71]. In two species of cyprinidontiform fishes, male and female parentals mate readily with hybrids [45, 72, 73], while mice discriminate against them [74]. This suggests that the likelihood of this process will depend in part on the biology of the hybridizing species.

An additional consideration is that hybrid reproductive isolation is most likely to evolve during a particular window of divergence between parental species. When the fitness of hybrid populations is low (i.e. corresponding to high levels of divergence between parental species), they are more prone to extinction or displacement by parentals (S6 Fig., S5 Text). This suggests that the evolution of hybrid reproductive isolation through this mechanism is most likely to occur in a period of evolutionary divergence during which species have accumulated some hybrid incompatibilities but have not diverged to the point at which hybrids are largely inviable. The most detailed work characterizing genetic incompatibilities has been between *Drosophila* species, where hybrids generally have substantially reduced fitness compared to parents [56, 57, 75]. Hybrids between several other species studied to date, however, are affected by fewer incompatibilities or incompatibilities of weaker effects [26, 55, 59, 76–79]. Such groups may be more likely to form hybrid populations, and should be the focus of future empirical research. In addition, even species that currently have strong isolation may have historically produced hybrid populations, though investigating ancient hybrid speciation by the mechanism we describe would be challenging. This is because if parental and hybrid lineages have diverged substantially since the time of initial hybridization it may not be possible to determine whether or not incompatibilities were initially derived from parental genomes.

It is interesting to note that reduced frequency of reproductive isolation with increasing selection on hybrids can be mitigated to some extent by an increase in the total number of hybrid incompatibility pairs. In our simulations, we see a positive relationship between the number of interactions and the probability of developing reproductive isolation, and a negative relationship between the total strength of selection on hybrids and the probability of developing reproductive isolation (Figs. 3 and S6). This tradeoff suggests that reproductive isolation can evolve between hybrid and parental populations even when the fitness of hybrids is low (as in Figs. 3, 4, and S6, keeping in mind that extinction occurs frequently when hybrid fitness is nearly zero).

Similarly, our model is sensitive to skewed initial admixture proportions, but increasing the number of hybrid incompatibility pairs increases the probability that skewed hybrid populations will be isolated from both parental species by at least one incompatibility (S7 Fig.). For example, with two incompatibility pairs, the probability of isolation from both parental species in an ancestry-skewed population (65% parent 1) was 7% while with four incompatibility pairs the probability rose to 15%. In addition, because discrete populations in a cline often span a range of admixture proportions (e.g. [80–82]), it is likely that some hybrid populations will fall in the range where we predict that isolation can evolve. On the other hand, our results show that high levels of migration (as might be observed in continuous clines) can prevent isolation; future research should investigate the dynamics of this process in a range of hybrid zone structures.

Finally, our model assumes that coevolving incompatibilities or BDM incompatibilities arising from adaptive evolution frequently occur between species. Accumulating evidence suggests that incompatibilities arising from coevolution may be common [30, 36, 83–86]. For example, in marine copepods, coevolution between cytochrome *c* and cytochrome *c* oxidase results in a reciprocal breakdown of protein function in hybrids [86]. In addition, the fact that many known incompatibility genes involve sexual conflict, selfish genetic elements, or pathogen defense suggests an important role for coevolution in the origin of incompatibilities [36, 83, 87, 88]. Our model also applies to BDM incompatibilities that arise due to within-lineage adaptation, assuming that the fitness advantage of the derived alleles is not dependent on the parental environment. It is currently unknown whether incompatibilities are more likely to be neutral or adaptive. Though there is evidence for asymmetric selection on many hybrid incompatibilities [28, 29, 89], neutrality has not been established in these cases. Anecdotal evidence supports the idea that adaptive incompatibilities are common, since many of the genes underlying hybrid incompatibilities identified so far show evidence of positive selection within lineages [90], but the relative frequency of adaptive and neutral BDM incompatibilities awaits answers from further empirical research. Intriguingly, theoretical work also suggests that neutral BDM incompatibilities are unlikely to persist if there is gene flow between species [32].

The patterns predicted by our model are testable with empirical approaches. A large number of studies have successfully mapped genetic incompatibilities distinguishing species [25, 26, 41, 56, 57, 79, 91]. Ancestry at these sites can be determined in putative hybrid species, and the relative contribution of parental-derived incompatibilities to reproductive isolation can be determined experimentally. For some species, it may be possible to evaluate the dynamics of incompatibilities relative to the genetic background in experimentally generated hybrid swarms [92]. We predict that many hybrid populations exhibiting postzygotic isolation from parental species will have fixed incompatibility pairs for each parental species. Several cases of hybrid speciation report reduced fitness of offspring between parental and hybrid species consistent with the mechanism described here [6, 16, 53, 93] and are promising cases for further empirical research. Strikingly, a recent study on Italian sparrows concludes that reproductive isolation between parental and hybrid species is partly due to the fixation of parental-derived incompatibilities [94].

An intriguing implication of our model is that independently formed hybrid populations between the same parental species can develop reproductive isolation from each other. The likelihood of this outcome increases with the number of incompatibility pairs. In sunflowers, empirical studies of ecologically-mediated hybrid speciation have identified multiple hybrid species derived from the same parental species [95]. It is interesting to note that selection against hybrid incompatibilities could generate the same pattern in replicate hybrid populations. In fact, this mechanism could generate a species phylogeny pattern similar to that expected from an adaptive radiation, with multiple closely related species arising in a relatively short evolutionary window. This finding is striking because our model does not invoke adaptation and suggests that non-adaptive processes (i.e. selection against incompatibilities) could also explain clusters of rapidly arising, closely-related species.

## Methods

### Mathematical model of selection on hybrid incompatibilities

To characterize evolution at hybrid incompatibility loci in hybrid populations without drift, we used the equations described by Karlin and others [46, 47] to calculate changes in allele frequency as a result of two-locus selection. The frequency of gamete *i* at generation *t* is given by

$$x_{i}(t)=x_{i}(t-1)\frac{w_{i*}}{\overset{-}{w}}+\epsilon_{i}rD\frac{w_{14}}{\overset{-}{w}},$$ (1)

where *ε*_1_ = *ε*_4_ = -*ε*_2_ = -*ε*_3_ = -1, the marginal fitness of allele *i*,

$$w_{i*}=\sum_{j=1}^{4}x_{j}w_{ij}{}_{\;},$$ (2)

the mean fitness of the population,

$$\overset{-}{w}=\sum_{i=1}^{4}\sum_{j=1}^{4}x_{i}x_{j}w_{ij},$$ (3)

*w*_14_is the fitness of a double heterozygote, *r* is the recombination rate and *D* is linkage disequilibrium between the two loci. These equations assume random mating, non-overlapping generations and that fitness depends only on two-locus genotype and not on whether the chromosome was maternally or paternally inherited (i.e. *w*_*ij*_ = *w*_*ji*_). To model changes in allele frequencies over time, we developed a custom R script (available from github: https://github.com/melop/twolocusmodel). Iterating through the change in allele frequencies each generation as a result of selection gives the expected patterns of fixation at incompatibility loci without genetic drift (S4 Fig.; see also [48]).

The deterministic two-locus model of fixation of hybrid incompatibilities does not realistically predict expected patterns in natural populations because even large populations will have some level of genetic drift. To model drift, we added multinomial sampling of N diploid individuals and recalculated allele frequencies each generation (available from github: https://github.com/melop/twolocusmodel). Patterns of fixation incorporating genetic drift through multinomial sampling show similar dynamics to the model lacking genetic drift, with the exception of several equilibrium states specific to the latter (see S5 Fig., S2 Text).

### Description of simulation program

Exact results for more than two loci have proven difficult to obtain [96–99]. As a result, we developed a custom c++ program, called admix’em (github: https://github.com/melop/admixem), to simulate more complex scenarios. The code allows one to specify the number and length of chromosomes and the genomic locations of hybrid incompatibilities and neutral markers. The current implementation assumes non-overlapping generations and diploid sexual individuals. When modeling linkage, we assume a uniform recombination rate and one recombination event per chromosome per meiosis. Unless otherwise specified, we model all pairs of hybrid incompatibility loci as unlinked. As we are interested in short-term dynamics, we do not implement mutation.

Selection coefficients are assigned to particular allelic combinations according to a hybrid fitness matrix (see S1 and S2 Figs). Based on each individual’s genotype at the hybrid incompatibility loci, we calculate total individual fitness *w*, defined as the probability of survival of that individual. Total fitness across multiple incompatibility pairs is assumed to be multiplicative. Each female mates with one randomly selected male (but we also accommodate assortative mating, see S6 Text), and produces a Poisson distributed number of offspring with a mean = 2. After selection, if the carrying capacity (N) is not reached, additional offspring from the same mating events are drawn from a Poisson distribution with a new mean = (carrying capacity—current population size)/number of females. This process is repeated until carrying capacity is reached or females have no available gametes (set to a maximum of 10). A potential concern with this approach for maintaining a constant population size is that it could artificially preserve a hybrid population that would otherwise be ephemeral by continuing to sample offspring (up to 10 per female in our simulations). However, because parentals are present in each population (see below, at 50% frequency each parental species in the initial population), this allows for out-competition of hybrids by parentals when hybrid fitness is low.

All reported results are based on 500 replicate simulations, which were conducted for 2000 generations. In the majority of simulations (except S3 and S4 Texts) the hybrid population is initially colonized by 500 individuals of each parental species. Hybrid and parental populations were modeled as spatially distinct with migration parameters between them; most simulations specified one hybrid population formed between two parental populations (S3 Fig.) but we also simulated a stepping-stone model and a model with multiple independently formed hybrid populations (S6 Text, S13–S14 Figs). In simulations with migration, the number of migrating individuals each generation was determined by drawing from a binomial distribution with a mean equal to the number of migrating individuals. Details on individual simulations and results can be found in the supporting text. Hybrid populations are considered to have evolved reproductive barriers from both parental species if they fix at least one incompatibility from each parental type; the strength of reproductive isolation between hybrids and parental species will depend on the selection coefficient and number of incompatibilities.

## Supporting Information

S1 Text Models of hybrid incompatibility. (DOCX)

S2 Text The two-locus model with genetic drift and comparison to population simulations. (DOCX)

S3 Text Evaluation of the hybrid population reproductive isolation model under a range of parameters. (DOCX)

S4 Text Incompatibilities that do not result in reproductive isolation in the absence of strong drift. (DOCX)

S5 Text Validation of the model under a range of genetic architectures. (DOCX)

S6 Text Validation of the model with a range of demographic scenarios. (DOCX)

S7 Text Simulations of the inversion model of hybrid speciation. (DOCX)

S1 Fig Evolution of two-locus BDM incompatibilities and the fitness of hybrid genotypes. (A) One of two possible mutational paths to the development of a two-locus BDM incompatibility (Not shown is the case where both mutations occur on one lineage). These incompatibilities can arise as the result of neutral fixation (w_parental_ = w_ancestral_) or as the result of adaptive evolution (w_parental_>w_ancestral_). (B) Potential selection patterns on hybrid genotypes between the two parentals (assuming w_parental_>w_ancestral_); genotypes corresponding to selection coefficients *s*_1_ and *s*_2_ are indicated in blue and red respectively. For BDM incompatibilities, *s*_1_ and *s*_2_ can be asymmetric, and in neutral BDM incompatibilities either *s*_1_ or *s*_2_ will equal zero. (C) Fitness of hybrid individuals with each genotype will depend on the intensity of selection (*s*_1_, *s*_2_) and dominance (*h*_*A*_, *h*_*B*_) at the two loci. We assume for simplicity that the fitness advantage of all derived genotypes (here, xB and Ax) is equal.
(JPG)

S2 Fig Evolution of two-locus coevolving incompatibilities and the fitness of hybrid genotypes. (A) One of two possible mutational paths to the development of a two-locus coevolved incompatibility. Not shown is the case where B_2_ precedes A (see S4 Text). (B) Potential epistatic interactions among hybrid genotypes. Incompatibilities corresponding to *s*_1_ and *s*_2_ are indicated in blue and red, respectively. (C) Fitness of hybrid individuals with each genotype will depend the intensity of selection (*s*_1_, *s*_2_) and dominance (*h*_*A*_, *h*_*B*_) at the two loci.
(JPG)

S3 Fig Schematic of hybrid population model used in simulations. The simplest model of hybrid speciation evolves in a hybrid swarm via fixation of parental genetic incompatibility pairs in opposite directions (see Fig. 1). *f* is the proportion of the hybrid (H) population colonized by parent 1 (P1), *m*_*1*,*2*_ denotes migration rates between the parental and hybrid populations over *n* generations.
(JPG)

S4 Fig Fixation of two-locus hybrid incompatibilities under deterministic selection (the deterministic two-locus model). The expected parent 1-derived allele trajectories for two unlinked hybrid incompatibilities under the deterministic two-locus model depend on starting admixture proportions (*f* = 0.3–0.7 shown here), dominance parameters (*h*), and the intensity of selection (i.e. *s*_1_, *s*_2_, see S1 and S2 Figs). The solid line tracks ancestry at locus 1 and the dashed line shows ancestry at locus 2. (A) Neutral BDM incompatibility pairs do not fix if *f* ≠ 0.5; at *f =* 0.5 they fix for a hybrid genotype pair that is not incompatible with either parental species (see S4 Text). When incompatibilities are codominant (B, C), the two incompatibility loci fix deterministically for the major parent. At certain values of *h* (D, E, F), fixation is less dependent on initial admixture proportions.
(JPG)

S5 Fig Fixation of two-locus hybrid incompatibilities with genetic drift. The expected patterns of fixation for a coevolving hybrid incompatibility (S2 Fig.) under the two-locus model depend on starting admixture proportions (*f* = 0.3–0.7 shown here), dominance parameters (*h*), and asymmetry in selection (*s*_1_ ≠ *s*_2_). The solid line shows ancestry at locus 1 of an incompatibility and the dashed line shows ancestry at locus 2 of an incompatibility. (A) Parent 1 allele trajectories predicted by the two-locus model for a given set of parameters. (B) Results for the same parameters incorporating multinomial sampling of 10,000 individuals at each generation. (C) Results for the same parameters incorporating multinomial sampling of 1,000 individuals at each generation. Patterns of fixation depend less on initial admixture proportions as drift increases. The equilibrium at *f* = 0.5 in the selection-only model is unstable in the presence of drift.
(JPG)

S6 Fig Probability of isolation with increasing selection on hybrids. With increasing selection on F_1_ hybrids between the parental species, the probability that hybrid populations will develop reproductive isolation from both parents decreases. However, reproductive isolation is more likely to evolve with a greater number of hybrid incompatibilities pairs (HI) when controlling for the total strength of selection against F_1_ hybrids. Error bars show two standard errors. Simulation parameters were *h* = 0.5, *s*_1_ = *s*_2_, *f* = 0.5, and N = 1,000.
(JPG)

S7 Fig The effect of initial admixture proportion on the probability of isolation. Proportion of hybrid populations developing isolation from both parents as a function of admixture proportions, dominance (*h*) and population size (two incompatibility pairs, *s*_1_ = *s*_2_) with two (A) and four (B) incompatibility pairs. Isolation occurs most frequently at equal admixture proportions, but can occur in ancestry-skewed populations, especially if the populations are small, there is variation in dominance, or larger numbers of incompatibility pairs. Error bars show two standard errors.
(JPG)

S8 Fig Hybrid incompatibility models that do not frequently result in reproductive isolation between hybrid and parental populations in the absence of drift. (A) When hybrid populations form at equal admixture proportions, the deterministic model predicts that neutral BDM incompatibilities will fix for the ancestral genotype in a two-lineage model (left) and a genotype that is compatible with both species in a one-lineage model (right). (B) In a coevolution scenario, certain mutation orders result in an identical fitness matrix to A and thus do not result in reproductive isolation in hybrid populations. In all cases depicted, mutations in lineage 1 could occur in lineage 2 and vice versa but the expected effects on isolation from parental species do not change.
(JPG)

S9 Fig The effect of population size on the probability of isolation due to neutral BDM incompatibilities. As drift increases, the proportion of hybrid populations isolated from parentals by fixation of neutral BDM incompatibilities increases. However, this process does not occur as rapidly as deterministic selection on other types of hybrid incompatibilities. Simulation parameters: two neutral BDMI pairs (S8 Fig.), *s* = 0.1, *f* = 0.5, *h* = 0.5 for 500 replicate simulations.
(JPG)

S10 Fig Linkage between incompatibility pairs. Linkage between incompatibility pairs can change the probability of hybrid populations evolving reproductive isolation (S6 Table). (A) In scenario 1, linkage between loci in the same incompatibility pair does not influence the frequency of hybrid populations evolving reproductive isolation. (B) In linkage scenario 2, linkage between loci in different incompatibility pairs significantly decreases the frequency at which hybrid populations evolve reproductive isolation. The probability of recombination between two sites is indicated as *r*.
(JPG)

S11 Fig Mating with parents reduces fitness of allopatrically evolving hybrid populations. (A) Change in average hybrid population fitness over time in a simulation of 20 incompatibility pairs with dominance and selection coefficients drawn from an exponential distribution (see S5D Text). (B) The same hybrid population with a one generation burst of migrants from parent 1 (4Nm_1_ = 400) at generation 300. (C) The same hybrid population with a one generation burst of migrants from parent 2 (4Nm_2_ = 400) at generation 300. Notably, hybrid populations have lower average fitness after gene flow with either parent, but recover rapidly.
(JPG)

S12 Fig Effect of asymmetry in migration on the probability of isolation from both parents. Proportion of hybrid populations evolving isolation from both parents as a function of asymmetry in migration rates from parental populations. When migration is highly asymmetric hybrid populations are less likely to evolve reproductive isolation from parental species. Simulation conditions: two incompatibility pairs, *h* = 0.5, *s*_*1*_ = *s*_*2*_ = 0.1, *f* = 0.5, N = 1000 for 500 replicate simulations.
(JPG)

S13 Fig Hybrid zone structure used in simulations. Model of hybrid zone structure used in simulations of complex hybrid zone structures (see S6B Text). This structure of a gradient of hybrid populations with ongoing gene flow from parental and other hybrid populations is similar to many naturally occurring hybrid populations.
(JPG)

S14 Fig A schematic model of multiple replicate hybrid populations. Hybrid zone structure used in simulations of reciprocal hybrid isolation (S6C Text).
(JPG)

S15 Fig The effect of initial admixture proportion on the probability of isolation under the underdominant inversion model. Proportion of hybrid populations developing isolation from both parents as a function of admixture proportion with two underdominant inversions. Simulation conditions: *s*_*1*_ = *s*_*2*_ = 0.05, N = 1000 for 500 replicate simulations. Error bars show two standard errors.
(JPG)

S1 Table Comparison of rates of fixation for the two-locus model with genetic drift and the simulation program at different population sizes. (DOCX)

S2 Table The effect of increasing selection on hybrids on the probability of and time to isolation. (DOCX)

S3 Table The effect of population size on the probability of and time to isolation. (DOCX)

S4 Table Effects of asymmetry in selection on the probability of isolation. (DOCX)

S5 Table The effect of variation in dominance. (DOCX)

S6 Table Effect of linkage between hybrid incompatibilities on the probability of and time to isolation. (DOCX)

S7 Table The effect of on-going and bursts of parental immigration on the probability of and time to isolation. (DOCX)

S8 Table Independently formed hybrid populations can evolve reproductive isolation from each other. (DOCX)

S9 Table Abundance of hybrids in several previously studied natural hybrid populations. (DOCX)

S10 Table Parental preferences for conspecifics reduce the frequency of hybrid reproductive isolation. (DOCX)

S11 Table The effect of increasing selection on hybrids on the probability of and time to isolation under the underdominant inversion model. (DOCX)
