# Supplementary material for: Reproductive Isolation of Hybrid Populations Driven by Genetic Incompatibilities
Source: PLoS Genet. 2015 Mar 13;11(3):e1005041. doi: 10.1371/journal.pgen.1005041 (PMC4359097; doi:10.1371/journal.pgen.1005041)
Supplement: S1 Text — (DOCX) [file pgen.1005041.s001.docx]

**Text S1. Models of hybrid incompatibility**

In order to investigate changes in allele frequencies at hybrid incompatibility loci, we need to define explicit models of selection on each two-locus genotype. Different genotypes at hybrid incompatibility loci will experience different strengths of selection. This can be a consequence of dominance at each locus (*h*), the number of epistatic interactions, the order in which mutations occurred, and the relative fitness of the parental and ancestral genotypes.

Our simulations are based on fitness matrices representing adaptive BDM incompatibilities (Figure S1) and coevolving hybrid incompatibilities (Figure S2). Elsewhere, we discuss neutral BDM incompatibilities and coevolving hybrid incompatibilities that will not generate isolation in the absence of strong genetic drift (Figure S8). For simplicity, in most cases we simulate coevolving incompatibilities (Figure S2) and assume that selection on different epistatic interactions is symmetrical (*s*_1_=*s*_2_) and that all alleles are codominant (*h*=0.5). However, we relax these assumptions in Text S3C and D. Though we focus on coevolving incompatibilities (Figure S2) our results also generally apply to adaptive BDM incompatibilities which have a similar fitness matrix (Figure S1).

In Figures S1A and S2A, we show a single mutational path to each hybrid incompatibility type; however, in both cases, several possible mutational paths exist. Because the lineage and order in which mutations occur is expected to be random [24], we randomly assign a fitness matrix to each incompatibility in simulations (note: these matrices are identical at *h*=0.5 and *s*_1_=*s*_2_).

Using these fitness matrices and the model of two-locus selection developed by Karlin and others (hereafter the “deterministic two-locus model”, [46, 47]) we can determine the expected trajectory of incompatibility pairs in hybrid populations under different parameters (Figure S4). However, some of these dynamics differ in finite populations (see Text S2).
