# Supplementary material for: Reproductive Isolation of Hybrid Populations Driven by Genetic Incompatibilities
Source: PLoS Genet. 2015 Mar 13;11(3):e1005041. doi: 10.1371/journal.pgen.1005041 (PMC4359097; doi:10.1371/journal.pgen.1005041)
Supplement: S2 Text — (DOCX) [file pgen.1005041.s002.docx]

**Text S2. The two-locus model with genetic drift and comparison to population simulations.**

The deterministic two-locus model ([46]; see Methods) allows us to predict the patterns of fixation for different incompatibility types with different parameters (Figure S4; see also [48]) but is not realistic because even large populations will have some genetic drift, which is not accounted for in the model. To implement drift, we added multinomial sampling of N individuals at each generation (Figure S5). The major difference caused by implementing drift is that fixation patterns do not always follow the predictions of the deterministic two-locus model. This can be seen most clearly in Figure S5C, where some fixation trajectories differ from expectations under the deterministic two-locus model.

The two-locus model equations do not allow us to model the fates of more than one incompatibility pair. We thus wrote an explicit population simulation code (admix’em) described in the Methods. To validate this code, we compared results from simulations with admix’em to simulations of the two-locus model with drift implemented by multinomial sampling. We simulated a single coevolving hybrid incompatibility (*h*=0.5, *s*_1_=*s*_2_=0.1) with population size N=1,000 and 10,000. In both cases the results from admix’em and the two-locus model with drift are nearly identical (Table S1).
