# Supplementary material for: Reproductive Isolation of Hybrid Populations Driven by Genetic Incompatibilities
Source: PLoS Genet. 2015 Mar 13;11(3):e1005041. doi: 10.1371/journal.pgen.1005041 (PMC4359097; doi:10.1371/journal.pgen.1005041)
Supplement: S3 Text — (DOCX) [file pgen.1005041.s003.docx]

**Text S3. Evaluation of the hybrid population reproductive isolation model under a range of parameters**

Simulations presented in the main text demonstrate that with two hybrid incompatibility pairs, reproductive isolation frequently evolves (47±2%) due to fixation of hybrid incompatibility loci for both parents. This mechanism of hybrid isolation is most likely to be biologically relevant if it occurs under a broad range of parameters. We performed simulations of two coevolving hybrid incompatibility pairs (Figure S2) varying selection (*s*), dominance (*h*), diploid population size (N), admixture proportions (*f*), and degree of asymmetry in selection (*s*_1_≠*s*_2_). All results are based on 500 replicate simulations. We consider hybrid populations “isolated” from both parents if they fix for at least one incompatibility pair from each parent; the strength of isolation will always depend on the strength of selection on each incompatibility and the number of incompatibilities. Together, these simulations demonstrate that hybrid reproductive isolation due to fixation of genetic incompatibilities can occur under a range of parameters.

1. *The strength of selection on hybrids.*

Variation in the strength of selection on hybrids can influence the likelihood of hybrid isolation under our model. We performed simulations with two coevolving hybrid incompatibility pairs with different strengths of selection (Table S2, Figure S6). We find that the probability of isolation decreases as total selection on hybrids increases. Lower probability of isolation at higher levels of selection on hybrids is the result of hybrid populations becoming dominated by parental individuals.

1. *Population size*

A number of models of speciation rely on small population sizes to achieve isolation from parental species [e.g. see Gavrilets and Hastings 1996]. One feature of our model is that it does not rely on small population sizes; in large populations incompatibility pairs fix by selection, whereas in small populations they fix by chance. As a result, the proportion of hybrid populations evolving isolation from both parents is not strongly dependent on population size (Table S3). This suggests that our model is applicable to a range of natural hybrid populations.

1. *Initial admixture proportions*

To determine how changing admixture proportions influences the number of hybrid populations evolving isolation, we varied starting admixture proportions from *f*=0.5-0.7. As expected, the probability of isolation from both parents decreased with increasing ancestry skew (Figure S7). Initial admixture proportions close to *f*=0.5 are most likely to result in isolation from both parental species. However, even in skewed populations, isolation from both parents was possible when assuming variation in dominance among alleles (for e.g. *h* =Uniform(0,1), Figure S7A), at lower population sizes (N=100, Figure S6A), or when simulating a larger number of incompatibility pairs (Figure S7B).

1. *Asymmetric selection on hybrid incompatibilities*

For simplicity, in most cases we simulate symmetric selection on hybrid incompatibilities. To introduce asymmetric selection on different hybrid genotypes we use different selection coefficients for distinct interactions in the coevolution model, *s*_1_ and *s*_2_ (Figure S2). When selection is asymmetrical (i.e. *s*_1_ ≠ *s*_2_), the two-locus model has similar properties to *s*_1_ = *s*_2_ as long as *s*_1_ and *s*_2_ are both greater than 0 (Figure S4). To investigate this in finite populations, we randomly assigned *s*=0.1 to *s*_1_ or *s*_2_ independently for two coevolving incompatibility pairs and set the value for the other selection coefficient to 0.2**s*, 0.3**s*, 0.4**s*, or 0.5**s*. In these simulations, reciprocal isolation still developed frequently (Table S4).

The adaptive BDM model (Figure S1) might result in even more extreme asymmetry in selection on hybrid genotypes [Gavrilets 1997; Unkless and Orr 2009; 38]. To approximate this scenario, we simulated adaptive BDMIs (Figure S1) and randomly assigned *s*=0.5 to *s*_1_ or *s*_2_ independently for two hybrid incompatibility pairs, and assigned *s*=0.02 to the other selection coefficient. In these simulations 36 ± 2% of populations became isolated from both parental species. In populations fixing incompatibility pairs from both parents, the strength of isolation from each parent species was similar. On average, the absolute fitness difference between backcross types (hybrid x parent 1 and hybrid x parent 2) was only 0.003. The average fitness of a hybrid x parent cross after hybrid populations fixed for parental incompatibilities was 0.87 (compared to 0.76 for an F1 cross between the two parent species).

1. *Dominance*

In the above simulations, we model codominant hybrid incompatibilities but varying dominance (*h*) does not have major effects on our conclusions. To demonstrate this, we simulated two scenarios: 1) we drew *h* from a uniform distribution, requiring *h* at each locus to add to 1 and 2) we randomly assigned *h* discrete values of 0 (recessive), 0.5 (co-dominant), or 1 (dominant), again requiring *h* to add to 1 at each locus. Both of these scenarios result in a similar probability of isolation from parental species (Table S5).

**Text S3 References**

Gavrilets S, Hastings A (1996) Founder effect speciation: A theoretical reassessment. Am Nat 147: 466-491.

Gavrilets S (1997) Hybrid zones with Dobzhansky-type epistatic selection. Evolution 51: 1027-1035.

Unckless RL, Orr HA (2009) Dobzhansky-Muller incompatibilities and adaptation to a shared environment. Heredity 102: 214-217.
