# Supplementary material for: Reproductive Isolation of Hybrid Populations Driven by Genetic Incompatibilities
Source: PLoS Genet. 2015 Mar 13;11(3):e1005041. doi: 10.1371/journal.pgen.1005041 (PMC4359097; doi:10.1371/journal.pgen.1005041)
Supplement: S4 Text — (DOCX) [file pgen.1005041.s004.docx]

**Text S4. Incompatibilities that do not frequently result in reproductive isolation in the absence of strong drift**

Several types of genetic incompatibilities, such as neutral BDM incompatibilities (or other scenarios that generate identical fitness matrices, Figure S8), are not predicted to contribute to reproductive isolation between hybrids and parentals by incompatibility selection (Figure S8). This is because hybrid incompatibility pairs either do not fix, or fix for a genotype that is compatible with both parents (based on the deterministic two-locus model, Figure S4A, Figure S8). Simulations of incompatibilities with these fitness matrices confirm this prediction; the most frequently fixed genotypes are not incompatible with either parent (47±2% of simulations) and in many simulations (38±2%) hybrid populations did not fix for a particular parental genotype at incompatibility loci within 2,000 generations (simulation parameters: N=1000, *s*=0.1, *f*=0.5, *h*=0.5).

Nonetheless, with strong genetic drift, genotypes incompatible with one of the parents can fix by chance, contributing to isolation, even under a neutral BDMI model. This can be seen in Figure S9, which shows the proportion of hybrid populations isolated from both parents as a function of population size. As population size decreases and drift increases, the proportion of hybrid populations isolating from both parents increases. However, the time to isolation is longer than in cases where fixation is driven by deterministic selection (by drift N=100: 264±143 generations, N=500: 1544±289 generations, N=1000: 1679±356 generations, compared to Table S3).
