# Supplementary material for: Reproductive Isolation of Hybrid Populations Driven by Genetic Incompatibilities
Source: PLoS Genet. 2015 Mar 13;11(3):e1005041. doi: 10.1371/journal.pgen.1005041 (PMC4359097; doi:10.1371/journal.pgen.1005041)
Supplement: S5 Text — (DOCX) [file pgen.1005041.s005.docx]

**Text S5. Validation of the model under a range of genetic architectures**

Because species are likely to be differentiated by a number of incompatibilities even in the early stages of speciation [Cutter 2012] we investigated how the number of incompatibility pairs and their genetic architecture affects the probability of evolving reproductive isolation.

- 1. *Number of incompatibility pairs*

Theoretically, increasing the number of incompatibility pairs should increase the probability that hybrid populations will become isolated from both parents (by at least one incompatibility). However, increasing the number of incompatibility pairs will reduce hybrid fitness and thus may also increase the probability that hybrids will be outcompeted by parental species. Simulating 3-6 unlinked coevolving incompatibility pairs (*f*=0.5, *h*=0.5, *s*_1_=*s*_2_=0.1-0.2, N=1000) increased the probability of isolation as a function of the number of pairs and selection coefficient (Figure 3, 49-91%). This result is notable because the probability of reciprocal isolation increases even with an increase in total selection on hybrids (see also Figure S6).

- 1. *Probability of reciprocal isolation in the presence of neutral BDM incompatibilities*

Though there is little data on fitness matrices for specific hybrid incompatibilities (reviewed in [30]), neutral BDM incompatibilities may be common [24]. Because of this, we determined the frequency of isolation between hybrids and parents in simulations including neutral BDM incompatibilities, where either *s*_1_ or *s*_2_ equals zero. We simulate two coevolving hybrid incompatibility pairs as described above but also include one linked or unlinked neutral BDM incompatibility pair. We find that the presence of BDM incompatibilities does not significantly decrease the probability of isolation between hybrid and parental populations (unlinked: 44 ± 2%, 1 cM between coevolving incompatibility and neutral BDM as in Figure S10B: 49 ± 2%, compared to 47 ± 2% with no neutral BDMIs). Interestingly, linkage between a neutral BDM incompatibility and a coevolving incompatibility can result in more frequent fixation of one of the parental genotypes at BDMIs (16± 2%, compared to 0.6% without linkage). In such cases, neutral BDMIs will contribute to isolation between hybrids and parental species.

- 1. *Linkage between incompatibility pairs*

Linkage between sites involved in hybrid incompatibilities could influence the frequency of isolation because ancestry at linked sites will be correlated. In order to investigate this we simulated two coevolving hybrid incompatibility pairs and varied genetic distance between loci (50 cM, 10 cM, and 1 cM; Figure S10). Some scenarios of physical linkage did not have a strong effect on the probability of isolation, while others reduced the probability of isolation (Table S6).

- 1. *More complex incompatibility architectures*

We have focused on very simple models that show that hybrid reproductive isolation can evolve in principle. However, empirical studies suggest that even closely related species are sometimes differentiated by dozens of incompatibilities that vary in their fitness effects [55, 59, 76,77, Payseur et al. 2004]. To investigate whether hybrid reproductive isolation evolves with more complex incompatibility architectures, we simulated a large number of incompatibilities with variation in selection, dominance, and asymmetry. We simulate 20 pairs of hybrid incompatibilities with a randomly chosen genomic position (chromosome 1-20, position 1-25000000 bp). Dominance is drawn from a uniform distribution (0-1) and selection coefficients (*s*_1_ and *s*_2_) are drawn from an exponential distribution with a mean of *s*=0.05. Because *s*_1_ and *s*_2_ are determined independently this introduces asymmetry in selection, and if *s*_1_ or *s*_2_ is close to 0 (i.e. on the order of 1/N), this generates an effectively neutral BDM incompatibility.

In these simulations, hybrid populations had a high probability of developing isolation from both parental species (95±1%). On average, backcrosses between parentals and individuals from the hybrid population had a fitness of 0.79±0.11 after 500 generations of selection on hybrid populations, compared to an average of 0.61±0.07 for F_1_ hybrids between the two parental species (Figures 3, S11). The average fitness of hybrids mating with other hybrids in the population was 0.97±0.02.

To investigate how the frequency of hybrid reproductive isolation changes with stronger selection in this more biologically realistic scenario, we repeated these simulations drawing *s* from an exponential distribution with a mean of 0.1 and 0.2. The average fitness of F_1_ hybrids between the two parental species in these cases was 0.38±0.09 and 0.12±0.06 respectively. In these cases the frequency of being isolated from both parents within 500 generations by at least one incompatibility was dramatically reduced to 56**±**2% and 1.4**±**0.5% respectively. These results demonstrate that hybrid reproductive isolation is unlikely to evolve by this mechanism when hybrid fitness is nearly zero.

**Text S5 References**

Cutter AD (2012) The polymorphic prelude to Bateson-Dobzhansky-Muller incompatibilities. Trends in Ecology & Evolution 27: 209-218.

Payseur BA, Krenz JG, Nachman MW (2004) Differential patterns of introgression across the X chromosome in a hybrid zone between two species of house mice. Evolution 58: 2064-2078.
