# Supplementary material for: Reproductive Isolation of Hybrid Populations Driven by Genetic Incompatibilities
Source: PLoS Genet. 2015 Mar 13;11(3):e1005041. doi: 10.1371/journal.pgen.1005041 (PMC4359097; doi:10.1371/journal.pgen.1005041)
Supplement: S6 Text — (DOCX) [file pgen.1005041.s006.docx]

**Text S6. Validation of the model under a range of demographic scenarios**

1. *Isolation with migration*

Hybrid populations in nature frequently experience ongoing gene flow with parental species. Given this, it is important to determine the impact of gene flow on how frequently hybrid populations evolve reproductive isolation. We simulated two migration scenarios, one with continuous migration from both parental populations (4N*m* = 8 – 20) and one with a single pulse of migration from either parent (migrating parent determined randomly) at 25, 50, and 75 generations in independent simulations respectively. Because populations with ongoing migration from both parents never completely fix for a particular genotype, we treated cases in which 95% of the hybrid population had a particular genotype as fixed. As expected, higher migration rates lowered the proportion of hybrid populations evolving isolation from both parents (Table S7, compared to 47± 2% with no migration). However, our results demonstrate that isolation still occurs frequently with fairly high levels of gene flow from the parental species. Notably, even before fixation, migration from parental populations lowers hybrid population fitness (Figure 4, Figure S11).

In the above simulations we assume symmetrical migration, but natural hybrid zones vary in the degree to which migration from parental populations is symmetrical (e.g. [60-62]). To explore how asymmetrical migration affects the probability of isolation we performed simulations varying the degree of asymmetry in migration from the parental populations (Figure S12). When migration is highly asymmetrical and migration rates are high, the probability of isolation is low but isolation still evolves at appreciable frequency in other scenarios (Figure S12).

*B. Migration in a stepping stone hybrid zone*

We use the simplest hybrid zone structure in our simulations (Figure S3), but hybrid zones often exist as a series of populations between the parental species. To simulate this, we modeled three hybrid populations between the parental populations with bidirectional migration between adjacent populations (Figure S13, Table S7). In this scenario, the hybrid population at the center of the hybrid zone (H2, Figure S13) experiences less direct gene flow from the parental populations, and should evolve reproductive isolation more frequently than those adjacent to the parental populations. As expected, we found that the hybrid population at the center of the hybrid zone developed isolation from parental species more frequently (Table S7), and that hybrid populations closer to the parentals were less likely to develop isolation (4*Nm*=8: 25±2 - 29±2%, 4*Nm*=12: 13±2 - 18±2, 4*Nm*=20: 1.0±0.6 - 1.2±0.6%).

*C. Reciprocal isolation of multiple hybrid populations*

One interesting prediction of our model is that independent hybrid populations that form from the same parental species could evolve reproductive isolation from each other if they fix for different parental alleles at incompatibility pairs. To investigate this, we simulated two hybrid populations formed from the same parental species (Figure S14). In cases where both hybrid populations in a simulation evolved isolation from the parents, we asked how frequently these populations were isolated from each other. When there was no gene flow between the hybrid populations, isolation evolved frequently between these populations (2 HI: 50 ± 5%, 3HI: 81 ± 4%, Table S8). However, when hybrid populations were connected by gene flow, this outcome was significantly less likely (Table S8). These simulations suggest that hybridization between the same parental species could generate multiple, reproductively isolated hybrid populations, but that this outcome is more likely when gene flow is low.

*D. Non-random mating of parents in hybrid zones*

In our simulations, mating is random, and pure parental individuals become less common as the number of generations since initial hybridization increases. However, if parentals mate assortatively, larger numbers will exist in the hybrid population, increasing the risk of out-competition of hybrids by parents. To investigate this, we implemented a mating rule in which migrating parentals mate exclusively with conspecifics. If a conspecific is not sampled in 25, 50, or 75 attempts in separate simulations respectively, the parental individual does not mate. With increasing parental effort to mate with a conspecific, hybrid populations evolved isolation less frequently (Table S9). This suggests that hybrid populations are more likely to be outcompeted if parentals have strong preferences against mating with hybrids.
