# Supplementary material for: Reproductive Isolation of Hybrid Populations Driven by Genetic Incompatibilities
Source: PLoS Genet. 2015 Mar 13;11(3):e1005041. doi: 10.1371/journal.pgen.1005041 (PMC4359097; doi:10.1371/journal.pgen.1005041)
Supplement: S7 Text — (DOCX) [file pgen.1005041.s007.docx]

**Text S7. Simulations of the inversion model of hybrid speciation**

One previous model of hybrid speciation, that we will call the “underdominant inversion” model [14,15,17], explicitly incorporated genetic mechanisms of speciation. In this model, parental species are distinguished by two underdominant inversions. Simulations of this model suggested that hybrid reproductive isolation is expected to be rare without positive selection on hybrid genotypes [14,17]. However, in both of these simulation efforts the authors modeled a spatially explicit hybrid population with a narrow contact zone, and either no spatial isolation between hybrids and parents [14] or high rates of migration from parental populations [17]. This specific population structure may have influenced results, which suggested that hybrid speciation was unlikely in the absence of strong positive selection (e.g. ecological selection ~0.5, [17]) or inbreeding [14].

We performed additional simulations to evaluate whether the inversion model can result in reproductive isolation of hybrid individuals without positive selection in the hybrid swarm scenario we model. Specifically, we simulated two unlinked underdominant inversions with selection only against genotypes heterozygous for the inversion, and assumed that fitness is multiplicative. As in simulations of the epistatic incompatibility model, we simulated 50-50 admixture proportions, random mating, and a diploid population size of 1000 for 500 replicate simulations.

Interestingly, we find that in a hybrid swarm scenario, inversions frequently fix for one of the two possible homozygous recombinant inversion genotypes (43±2% of simulations with *s_1_*=*s_2_*=0.05, Table S11). These barriers evolved rapidly, on average within 212±59 generations with *s_1_*=*s_2_*=0.05. Thus, the probability and time to isolation are quite similar to the epistatic incompatibility model (see Text S3). In addition, the probability of isolation decreases with increasing skew in admixture proportions, consistent with the results of our model (Figure S15 compared to Figure S7).

Our results imply that in a hybrid swarm that is spatially isolated from parental species, reproductive isolation can arise frequently and quickly from underdominant inversions without invoking positive selection. Results for our epistatic incompatibility model show that an increase in the number of incompatibility pairs results in an increase in the probability of isolation, even with stronger selection on hybrids (Figure 3, Figure S6). It will be interesting to explore whether the underdominant inversion model has similar dynamics, since the opposite trend was reported under the simulation conditions of the original paper [14].
