# Supplementary figures and images for: Reproductive Isolation of Hybrid Populations Driven by Genetic Incompatibilities
Source: PLoS Genet. 2015 Mar 13;11(3):e1005041. doi: 10.1371/journal.pgen.1005041 (PMC4359097; doi:10.1371/journal.pgen.1005041)

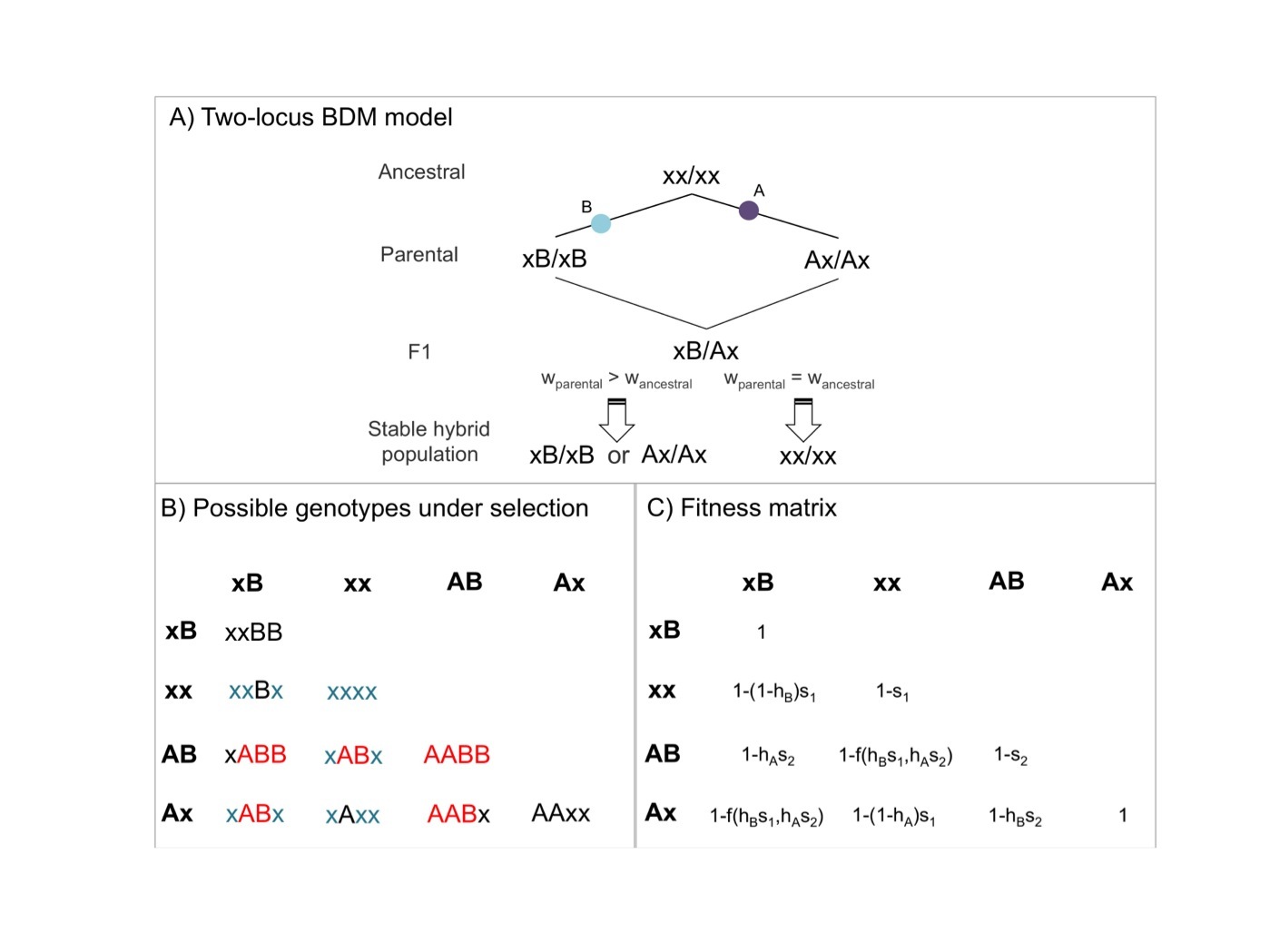

Supplement: S1 Fig — (A) One of two possible mutational paths to the development of a two-locus BDM incompatibility (Not shown is the case where both mutations occur on one lineage). These incompatibilities can arise as the result of neutral fixation (wparental = wancestral) or as the result of adaptive evolution (wparental>wancestral). (B) Potential selection patterns on hybrid genotypes between the two parentals (assuming wparental>wancestral); genotypes corresponding to selection coefficients s 1 and s 2 are indicated in blue and red respectively. For BDM incompatibilities, s 1 and s 2 can be asymmetric, and in neutral BDM incompatibilities either s 1 or s 2 will equal zero. (C) Fitness of hybrid individuals with each genotype will depend on the intensity of selection (s 1, s 2) and dominance (h A, h B) at the two loci. We assume for simplicity that the fitness advantage of all derived genotypes (here, xB and Ax) is equal. (JPG) [file pgen.1005041.s008.jpg]

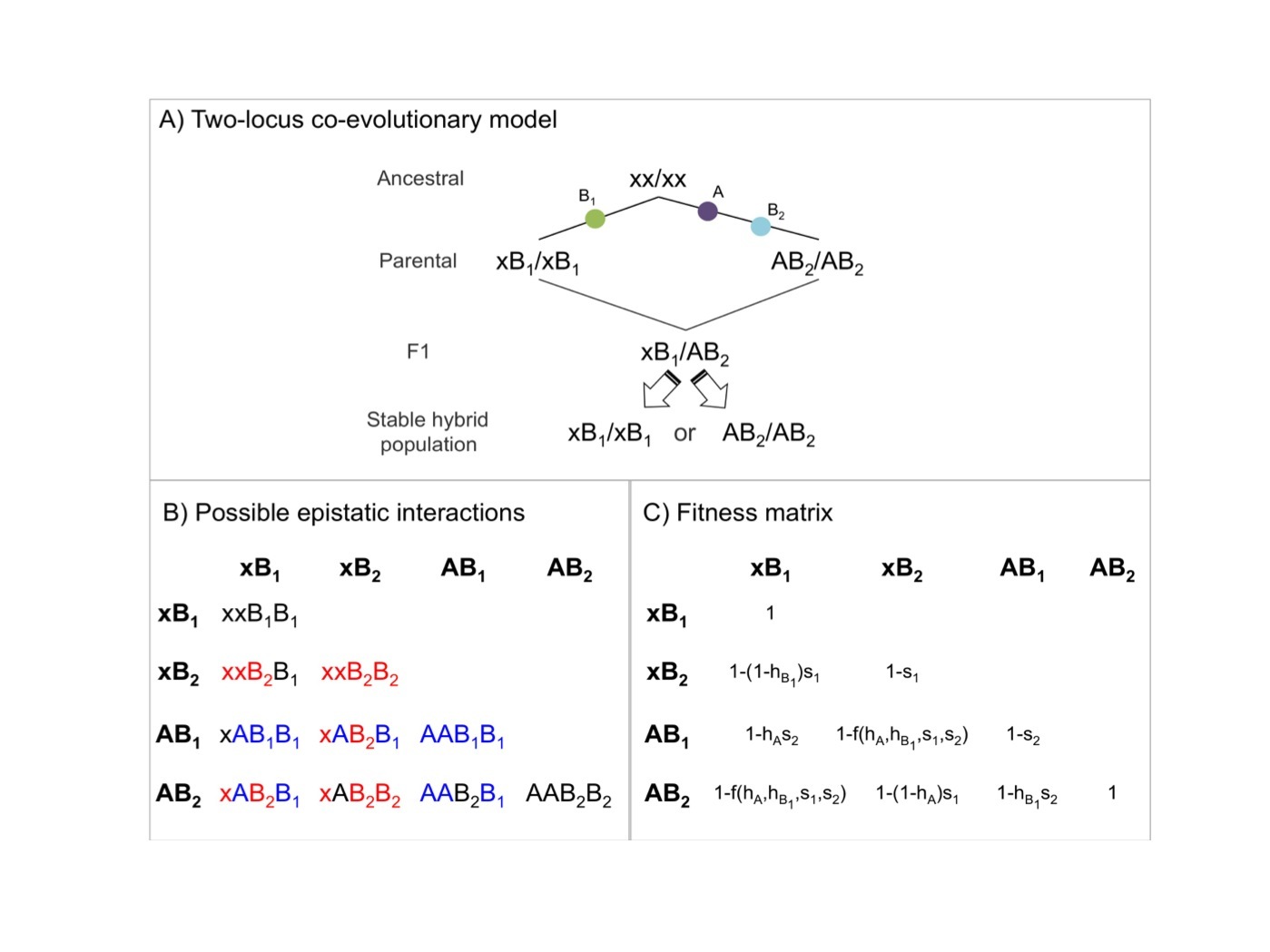

Supplement: S2 Fig — (A) One of two possible mutational paths to the development of a two-locus coevolved incompatibility. Not shown is the case where B2 precedes A (see S4 Text). (B) Potential epistatic interactions among hybrid genotypes. Incompatibilities corresponding to s 1 and s 2 are indicated in blue and red, respectively. (C) Fitness of hybrid individuals with each genotype will depend the intensity of selection (s 1, s 2) and dominance (h A, h B) at the two loci. (JPG) [file pgen.1005041.s009.jpg]

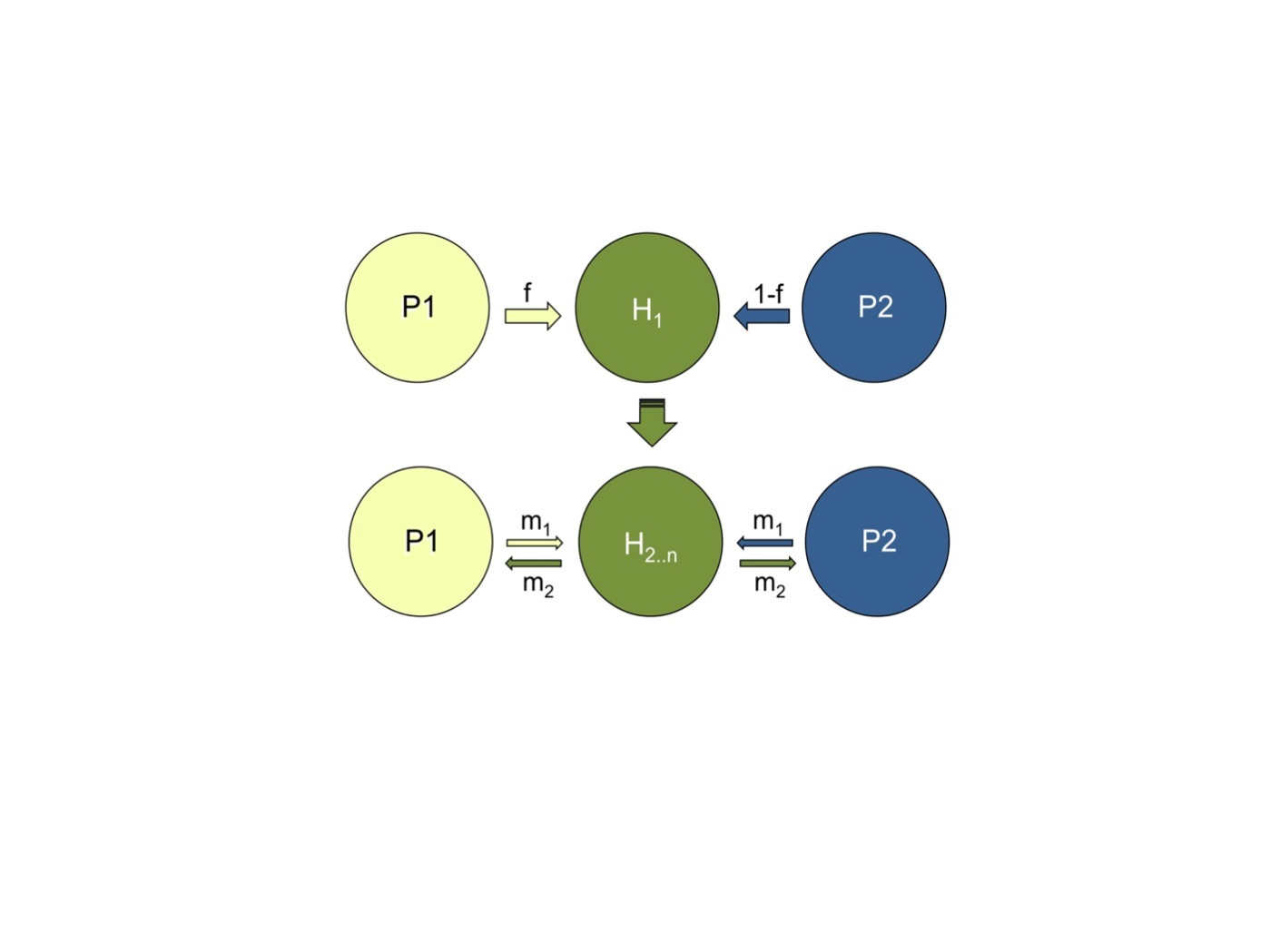

Supplement: S3 Fig — The simplest model of hybrid speciation evolves in a hybrid swarm via fixation of parental genetic incompatibility pairs in opposite directions (see Fig. 1). f is the proportion of the hybrid (H) population colonized by parent 1 (P1), m 1,2 denotes migration rates between the parental and hybrid populations over n generations. (JPG) [file pgen.1005041.s010.jpg]

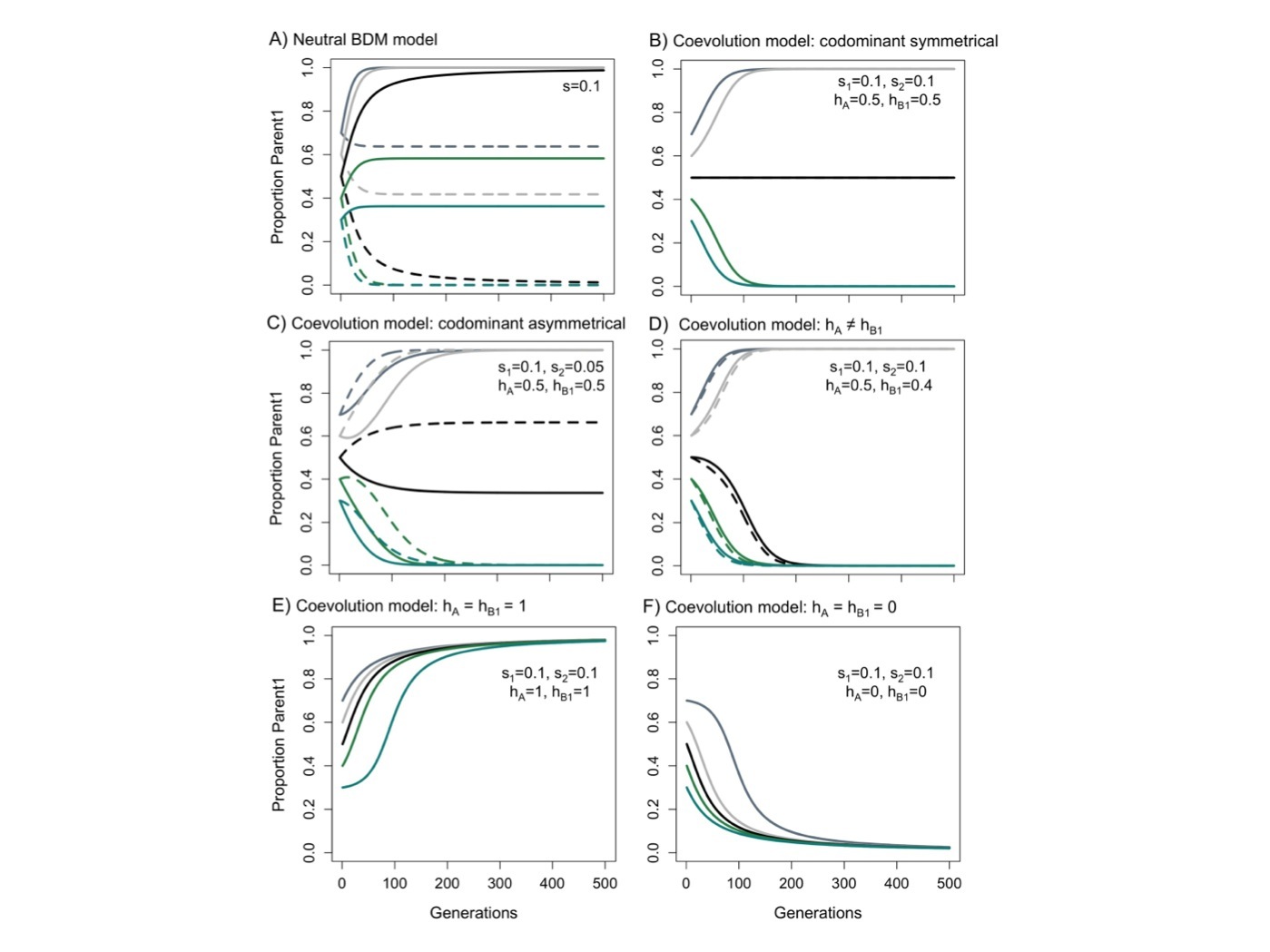

Supplement: S4 Fig — The expected parent 1-derived allele trajectories for two unlinked hybrid incompatibilities under the deterministic two-locus model depend on starting admixture proportions (f = 0.3–0.7 shown here), dominance parameters (h), and the intensity of selection (i.e. s 1, s 2, see S1 and S2 Figs). The solid line tracks ancestry at locus 1 and the dashed line shows ancestry at locus 2. (A) Neutral BDM incompatibility pairs do not fix if f ≠ 0.5; at f = 0.5 they fix for a hybrid genotype pair that is not incompatible with either parental species (see S4 Text). When incompatibilities are codominant (B, C), the two incompatibility loci fix deterministically for the major parent. At certain values of h (D, E, F), fixation is less dependent on initial admixture proportions. (JPG) [file pgen.1005041.s011.jpg]

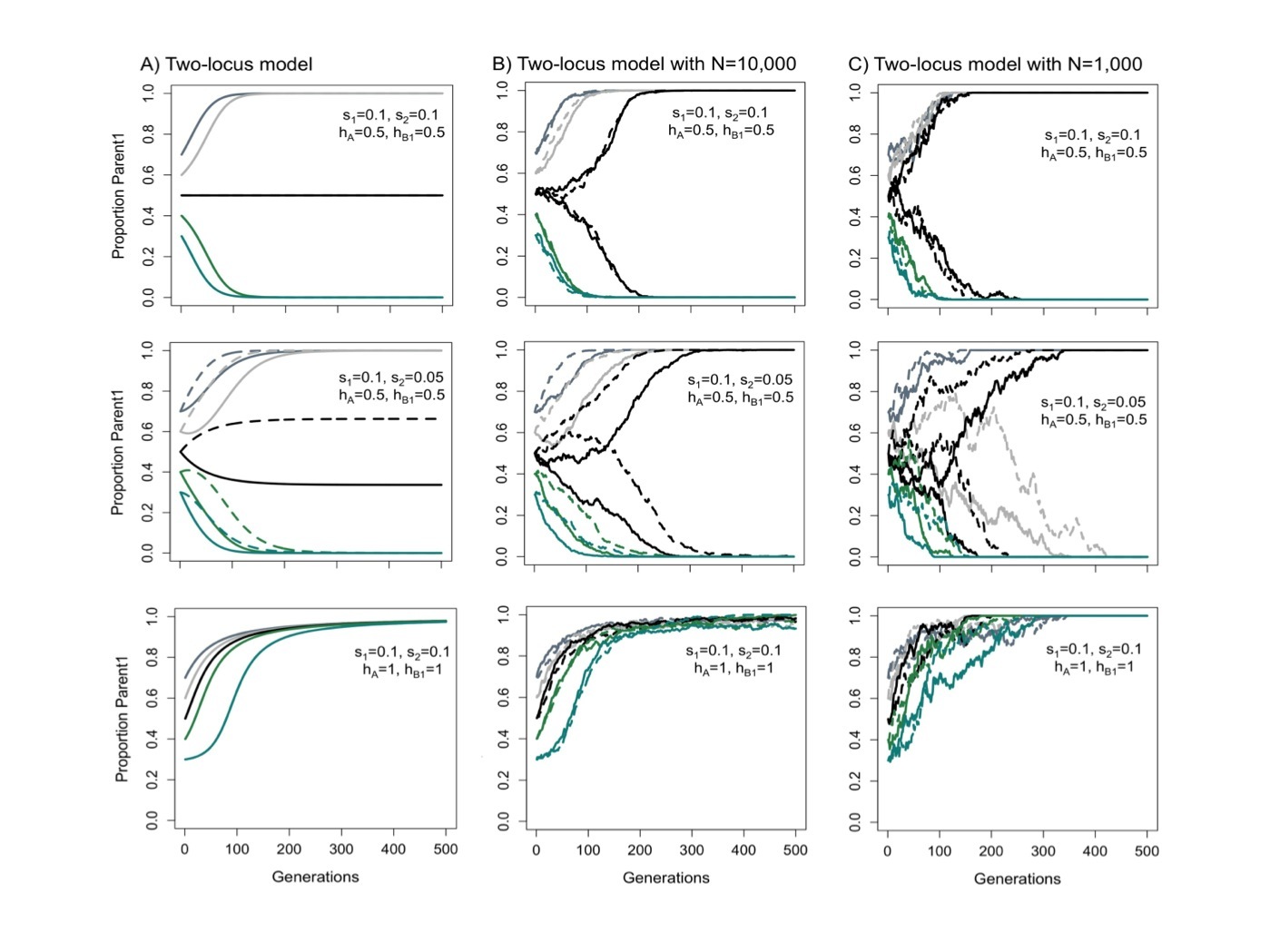

Supplement: S5 Fig — The expected patterns of fixation for a coevolving hybrid incompatibility (S2 Fig.) under the two-locus model depend on starting admixture proportions (f = 0.3–0.7 shown here), dominance parameters (h), and asymmetry in selection (s 1 ≠ s 2). The solid line shows ancestry at locus 1 of an incompatibility and the dashed line shows ancestry at locus 2 of an incompatibility. (A) Parent 1 allele trajectories predicted by the two-locus model for a given set of parameters. (B) Results for the same parameters incorporating multinomial sampling of 10,000 individuals at each generation. (C) Results for the same parameters incorporating multinomial sampling of 1,000 individuals at each generation. Patterns of fixation depend less on initial admixture proportions as drift increases. The equilibrium at f = 0.5 in the selection-only model is unstable in the presence of drift. (JPG) [file pgen.1005041.s012.jpg]

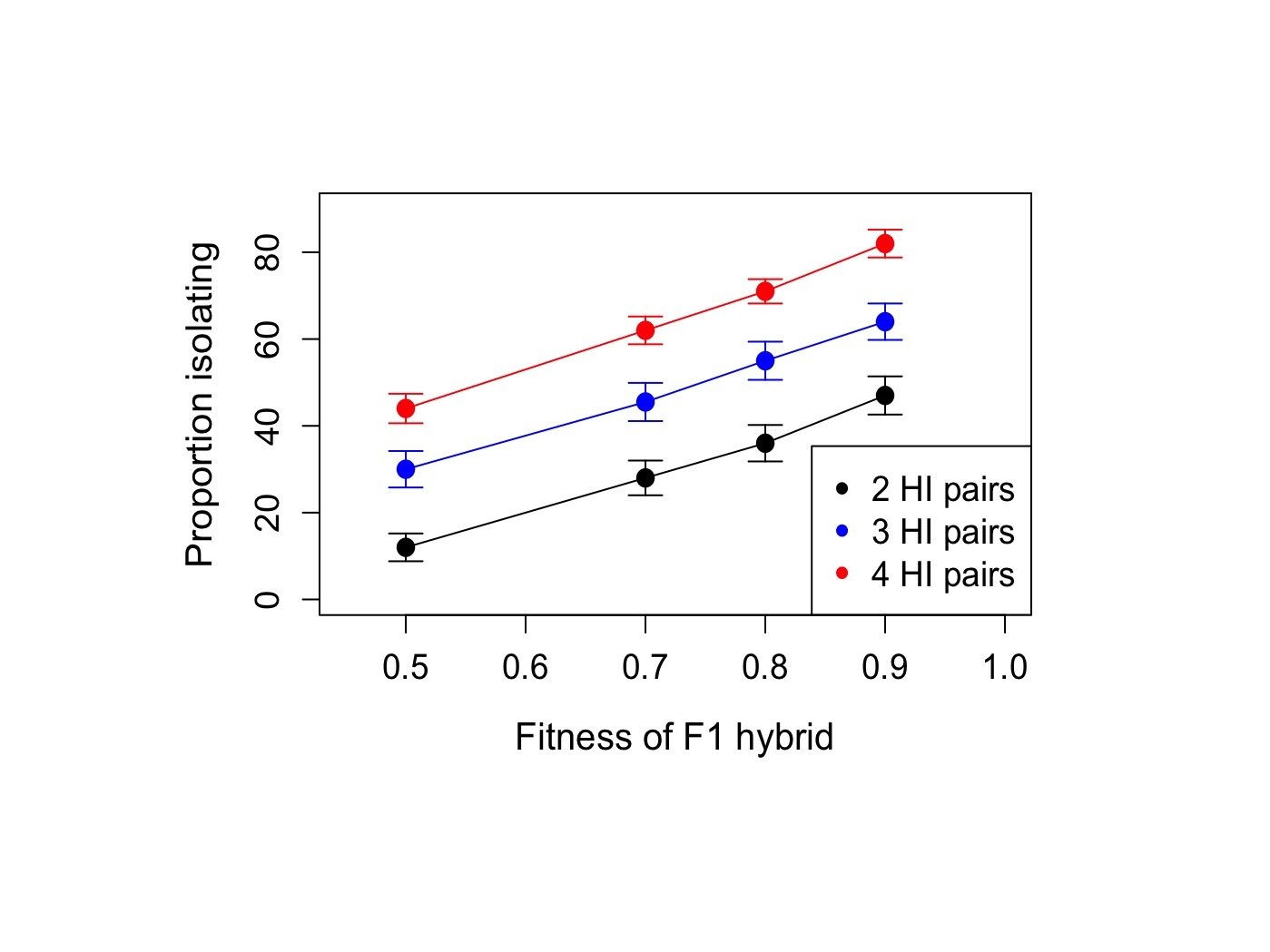

Supplement: S6 Fig — With increasing selection on F1 hybrids between the parental species, the probability that hybrid populations will develop reproductive isolation from both parents decreases. However, reproductive isolation is more likely to evolve with a greater number of hybrid incompatibilities pairs (HI) when controlling for the total strength of selection against F1 hybrids. Error bars show two standard errors. Simulation parameters were h = 0.5, s 1 = s 2, f = 0.5, and N = 1,000. (JPG) [file pgen.1005041.s013.jpg]

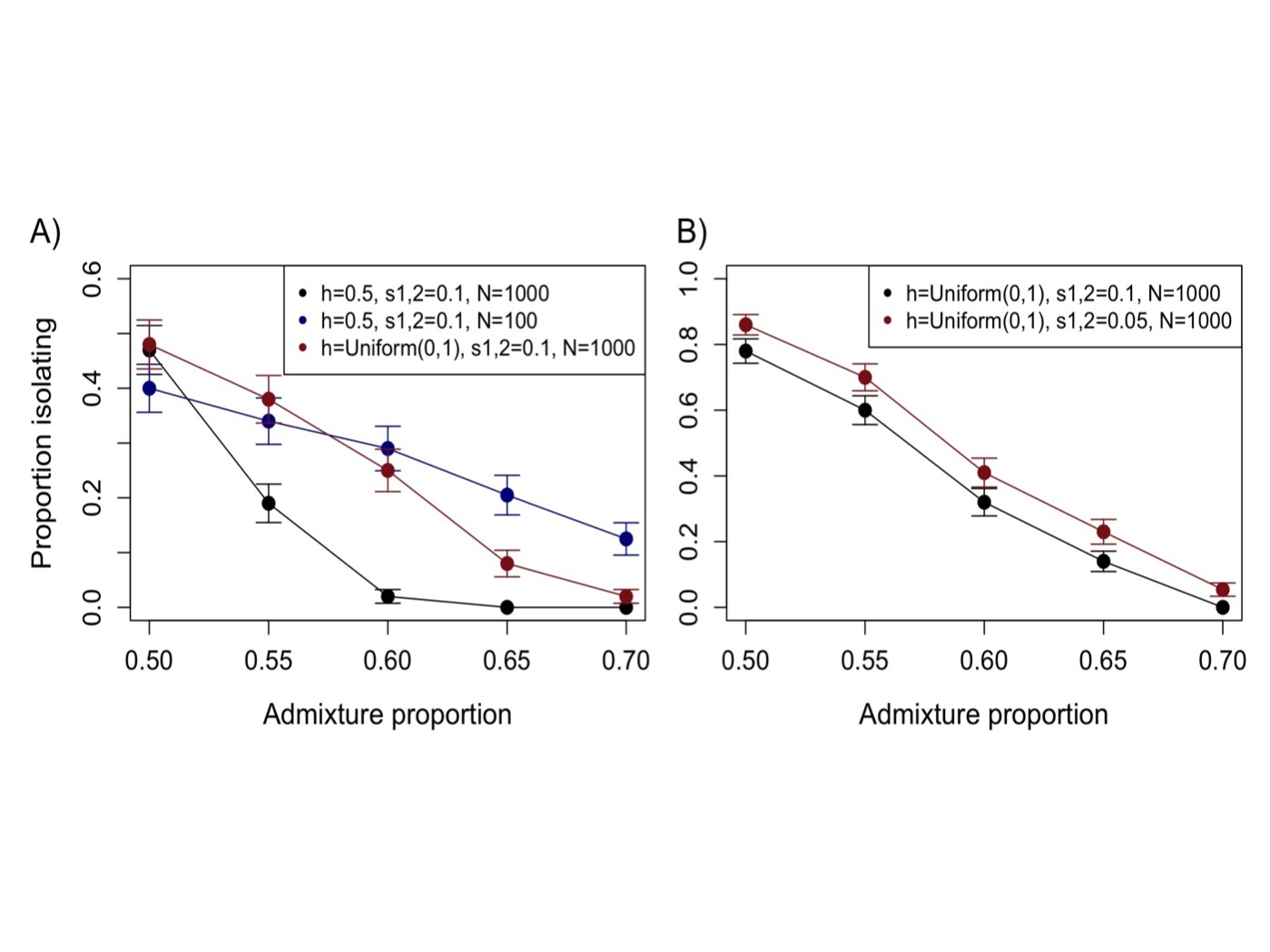

Supplement: S7 Fig — Proportion of hybrid populations developing isolation from both parents as a function of admixture proportions, dominance (h) and population size (two incompatibility pairs, s 1 = s 2) with two (A) and four (B) incompatibility pairs. Isolation occurs most frequently at equal admixture proportions, but can occur in ancestry-skewed populations, especially if the populations are small, there is variation in dominance, or larger numbers of incompatibility pairs. Error bars show two standard errors. (JPG) [file pgen.1005041.s014.jpg]

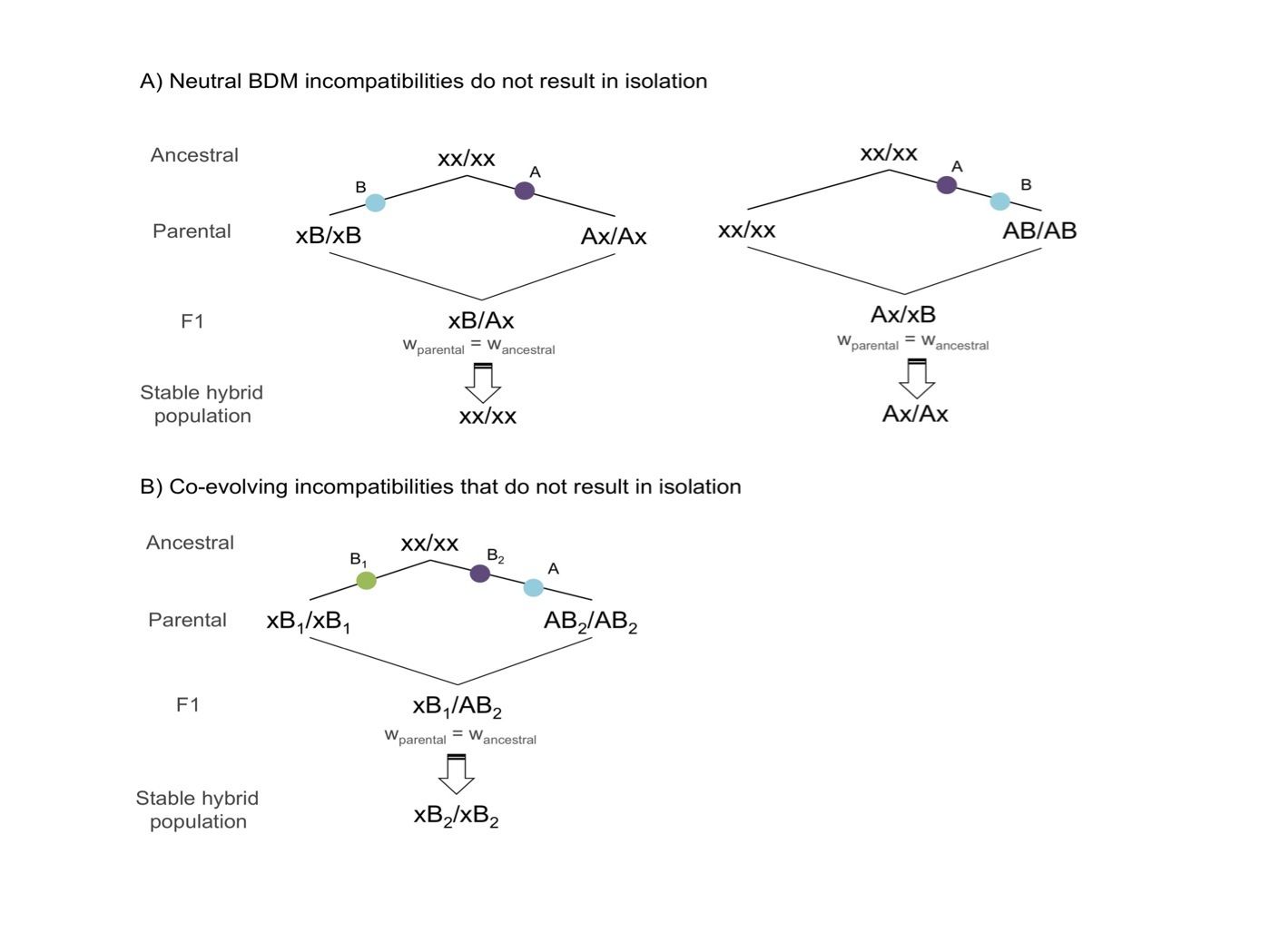

Supplement: S8 Fig — (A) When hybrid populations form at equal admixture proportions, the deterministic model predicts that neutral BDM incompatibilities will fix for the ancestral genotype in a two-lineage model (left) and a genotype that is compatible with both species in a one-lineage model (right). (B) In a coevolution scenario, certain mutation orders result in an identical fitness matrix to A and thus do not result in reproductive isolation in hybrid populations. In all cases depicted, mutations in lineage 1 could occur in lineage 2 and vice versa but the expected effects on isolation from parental species do not change. (JPG) [file pgen.1005041.s015.jpg]

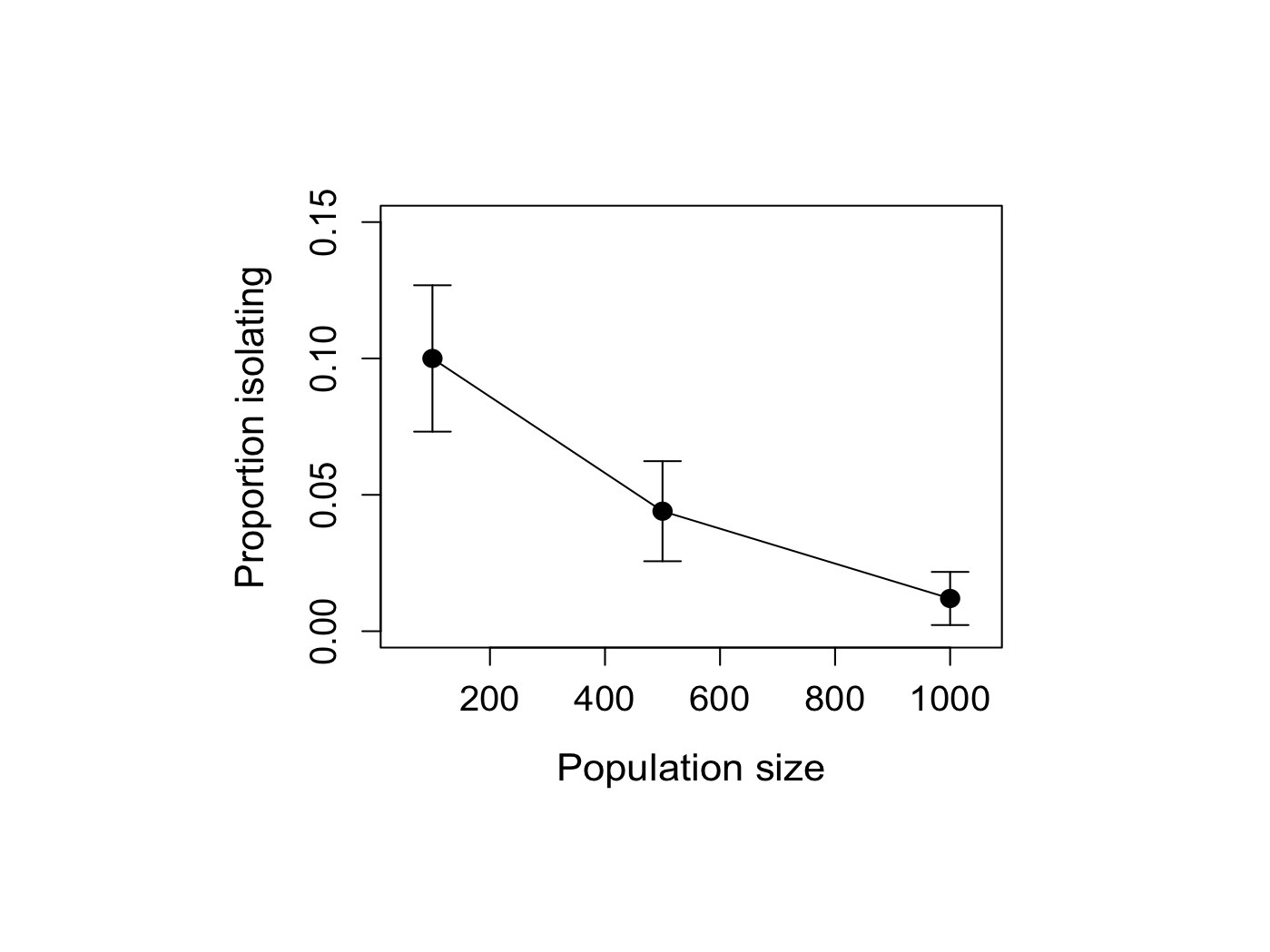

Supplement: S9 Fig — As drift increases, the proportion of hybrid populations isolated from parentals by fixation of neutral BDM incompatibilities increases. However, this process does not occur as rapidly as deterministic selection on other types of hybrid incompatibilities. Simulation parameters: two neutral BDMI pairs (S8 Fig.), s = 0.1, f = 0.5, h = 0.5 for 500 replicate simulations. (JPG) [file pgen.1005041.s016.jpg]

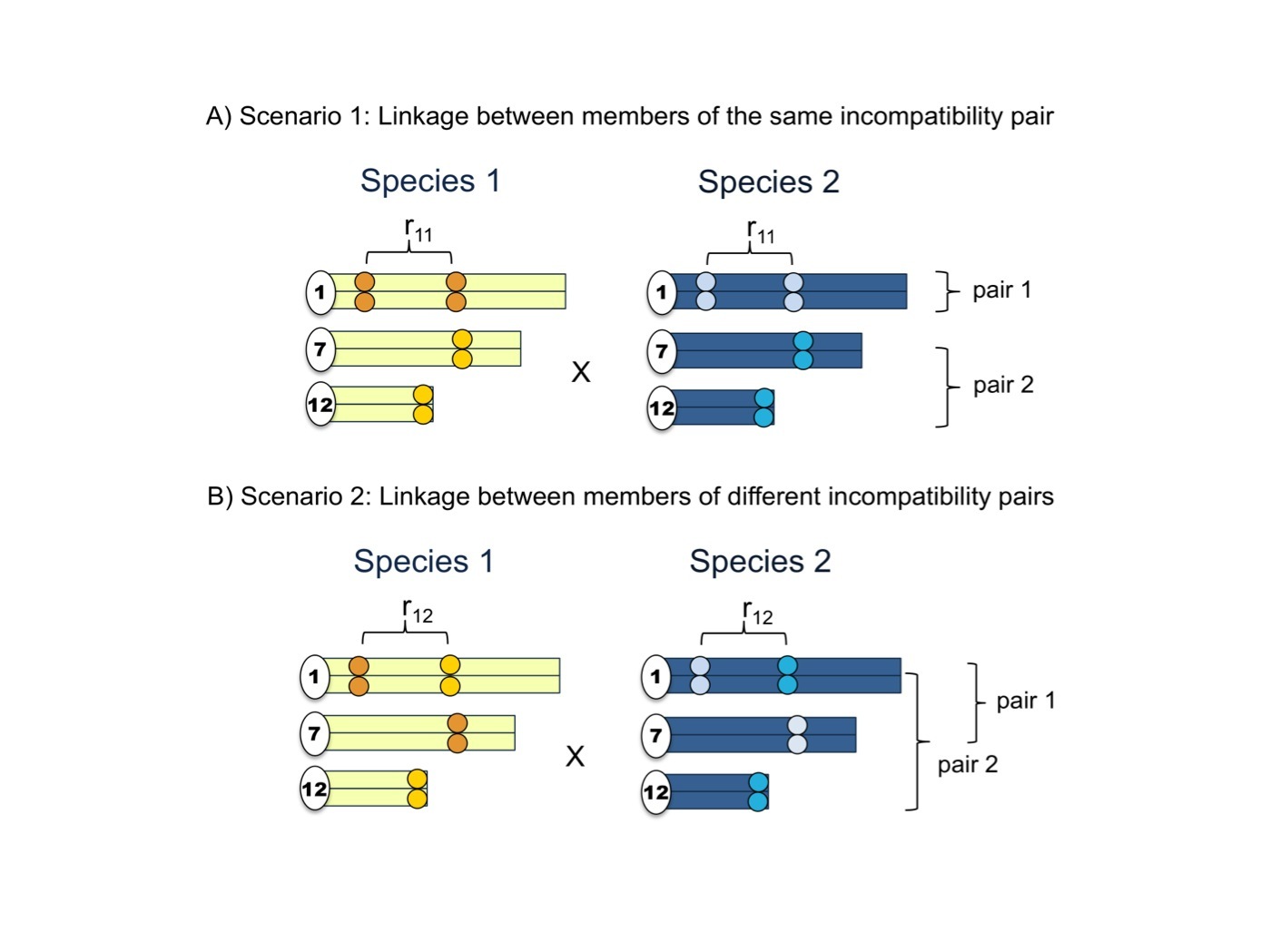

Supplement: S10 Fig — Linkage between incompatibility pairs can change the probability of hybrid populations evolving reproductive isolation (S6 Table). (A) In scenario 1, linkage between loci in the same incompatibility pair does not influence the frequency of hybrid populations evolving reproductive isolation. (B) In linkage scenario 2, linkage between loci in different incompatibility pairs significantly decreases the frequency at which hybrid populations evolve reproductive isolation. The probability of recombination between two sites is indicated as r. (JPG) [file pgen.1005041.s017.jpg]

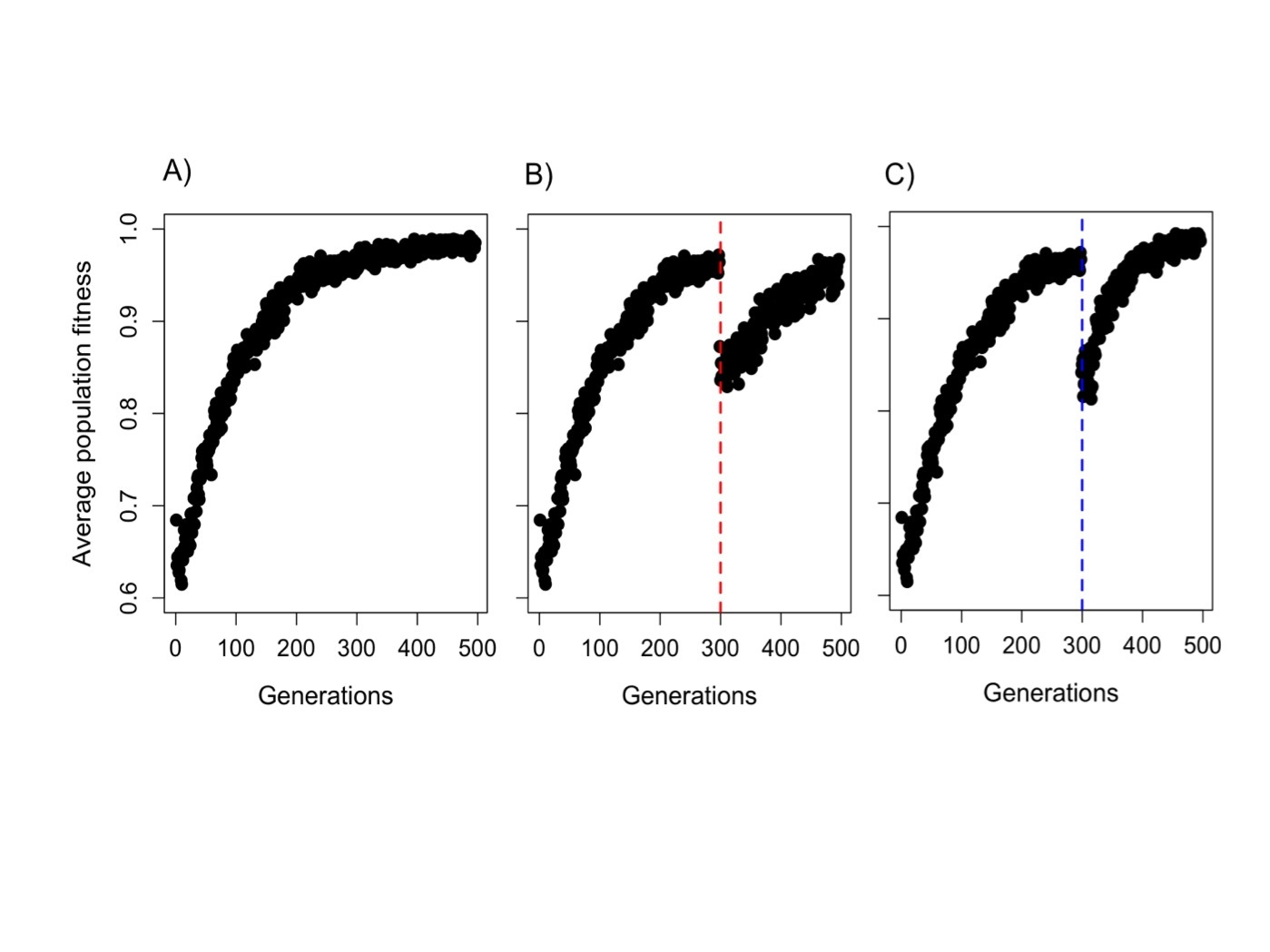

Supplement: S11 Fig — (A) Change in average hybrid population fitness over time in a simulation of 20 incompatibility pairs with dominance and selection coefficients drawn from an exponential distribution (see S5D Text). (B) The same hybrid population with a one generation burst of migrants from parent 1 (4Nm1 = 400) at generation 300. (C) The same hybrid population with a one generation burst of migrants from parent 2 (4Nm2 = 400) at generation 300. Notably, hybrid populations have lower average fitness after gene flow with either parent, but recover rapidly. (JPG) [file pgen.1005041.s018.jpg]

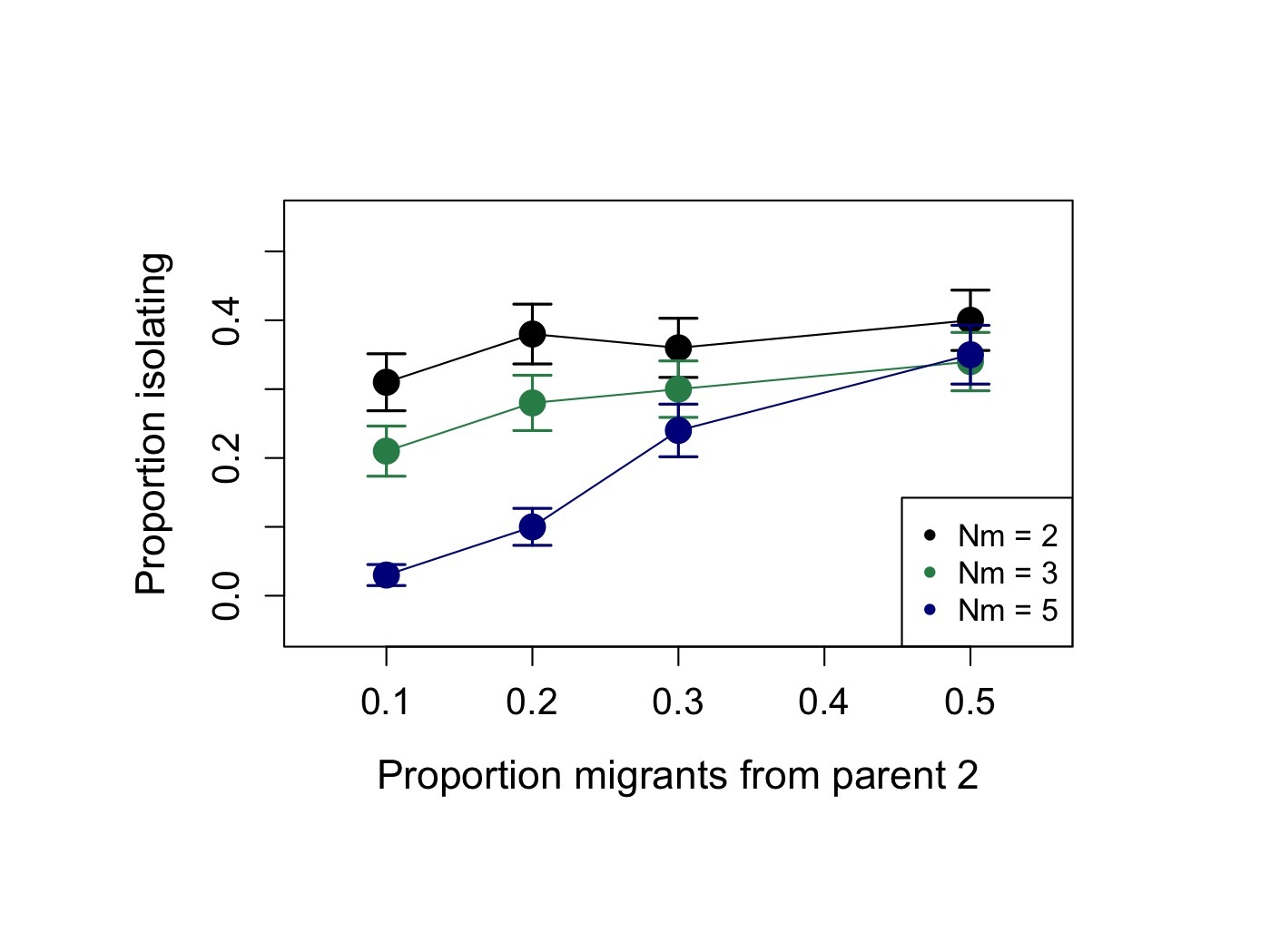

Supplement: S12 Fig — Proportion of hybrid populations evolving isolation from both parents as a function of asymmetry in migration rates from parental populations. When migration is highly asymmetric hybrid populations are less likely to evolve reproductive isolation from parental species. Simulation conditions: two incompatibility pairs, h = 0.5, s 1 = s 2 = 0.1, f = 0.5, N = 1000 for 500 replicate simulations. (JPG) [file pgen.1005041.s019.jpg]

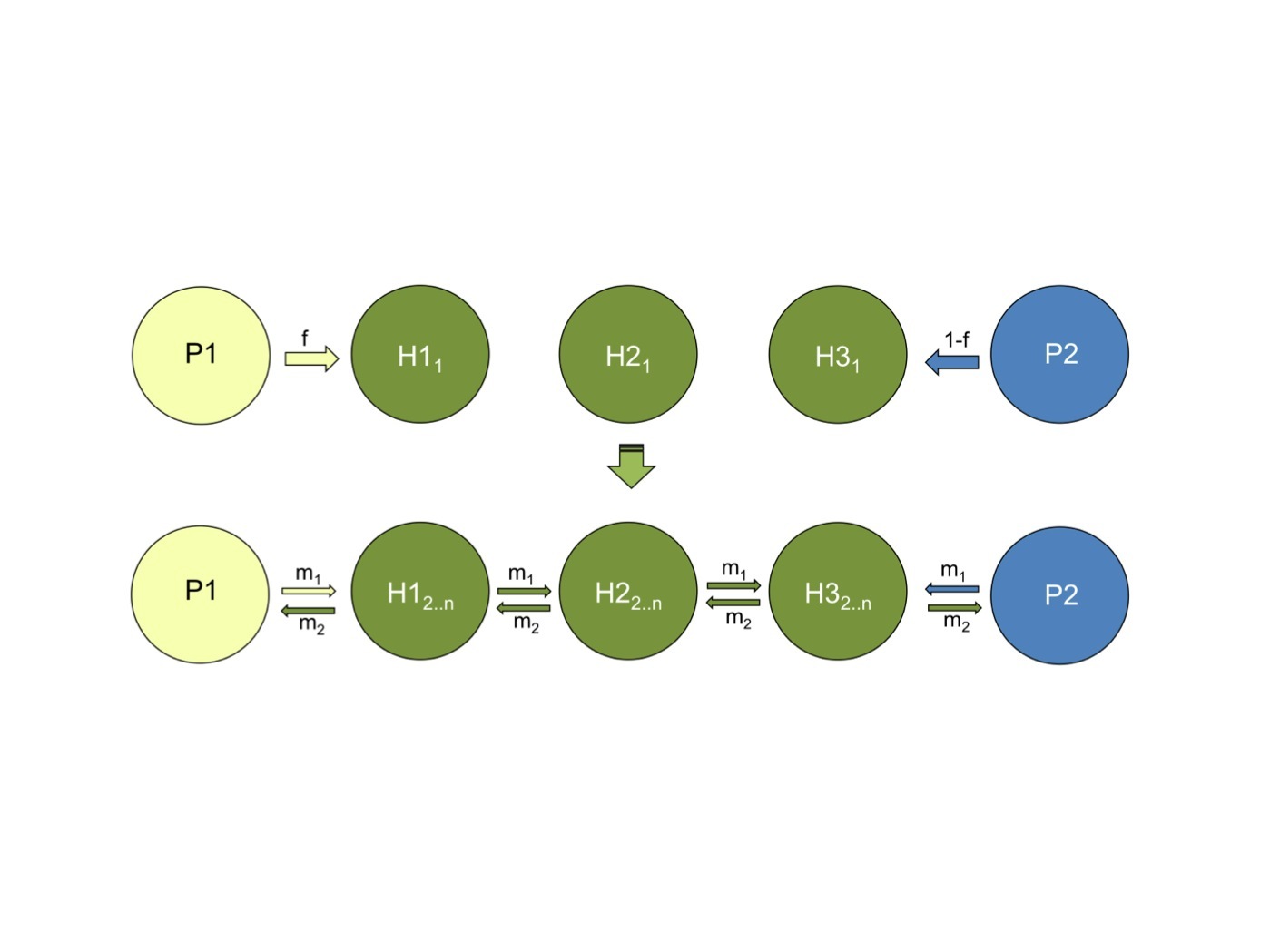

Supplement: S13 Fig — Model of hybrid zone structure used in simulations of complex hybrid zone structures (see S6B Text). This structure of a gradient of hybrid populations with ongoing gene flow from parental and other hybrid populations is similar to many naturally occurring hybrid populations. (JPG) [file pgen.1005041.s020.jpg]

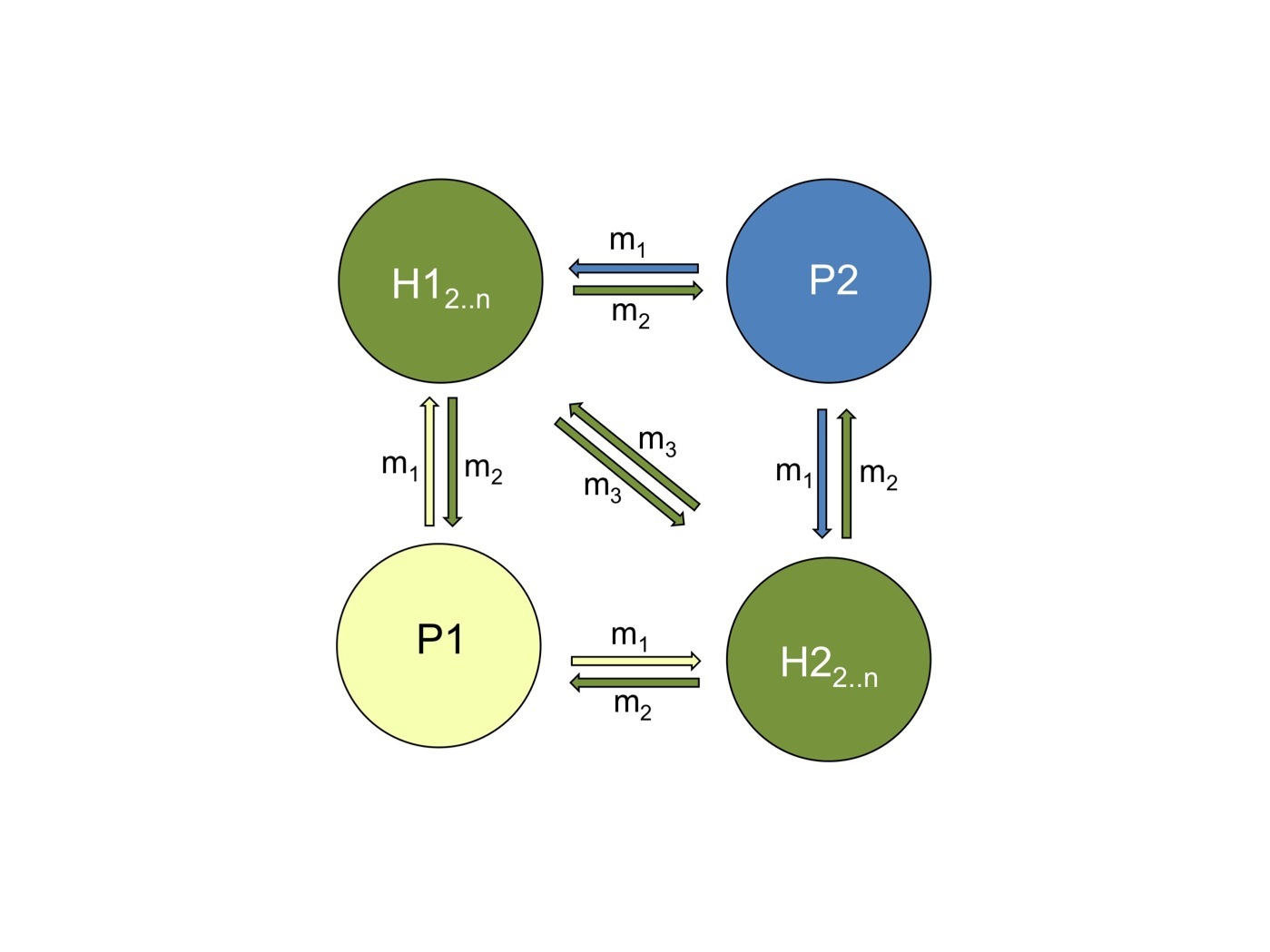

Supplement: S14 Fig — Hybrid zone structure used in simulations of reciprocal hybrid isolation (S6C Text). (JPG) [file pgen.1005041.s021.jpg]

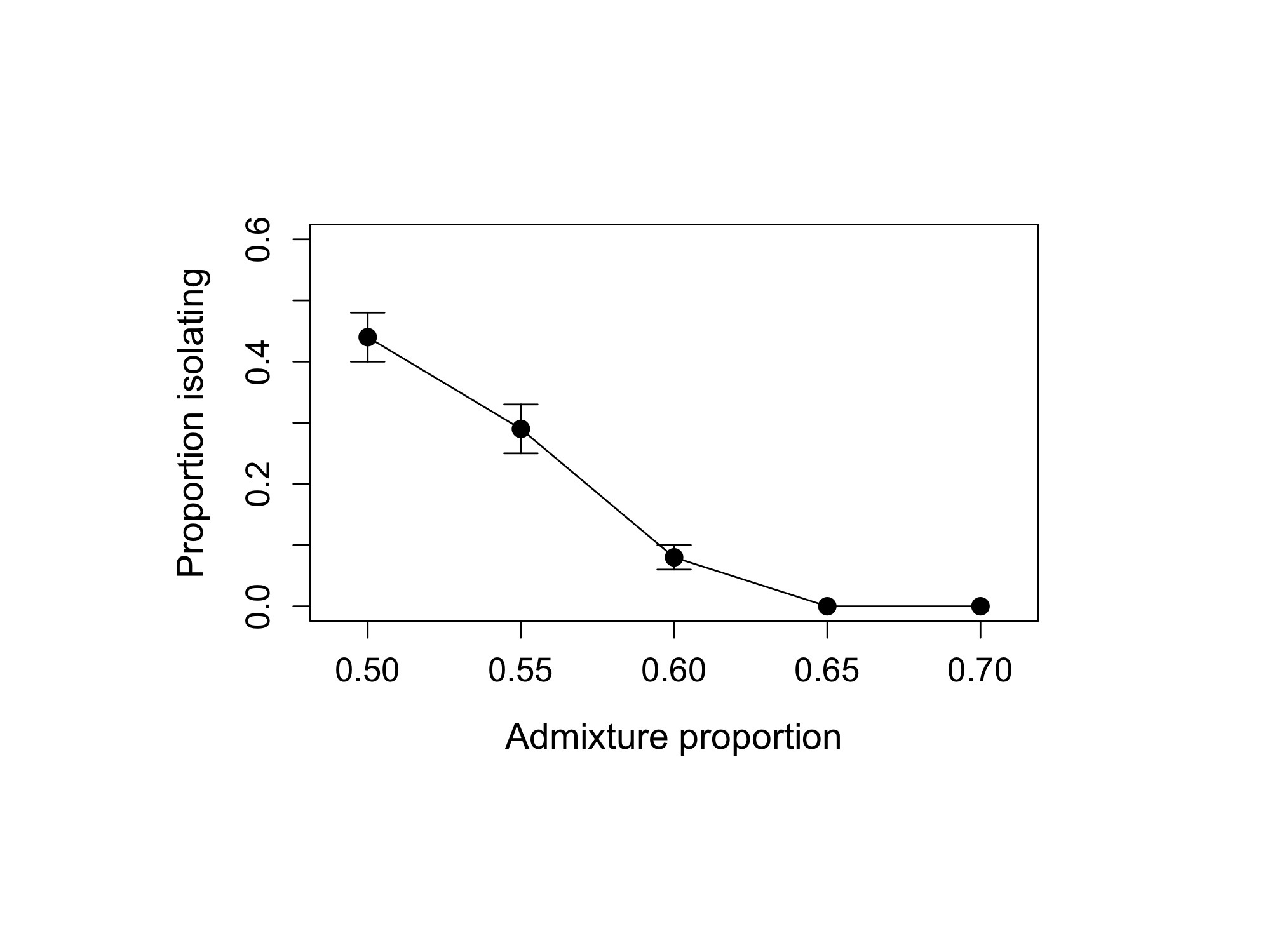

Supplement: S15 Fig — Proportion of hybrid populations developing isolation from both parents as a function of admixture proportion with two underdominant inversions. Simulation conditions: s 1 = s 2 = 0.05, N = 1000 for 500 replicate simulations. Error bars show two standard errors. (JPG) [file pgen.1005041.s022.jpg]
