# Supplementary material for: Reproductive Isolation of Hybrid Populations Driven by Genetic Incompatibilities
Source: PLoS Genet. 2015 Mar 13;11(3):e1005041. doi: 10.1371/journal.pgen.1005041 (PMC4359097; doi:10.1371/journal.pgen.1005041)
Supplement: S1 Table — (DOCX) [file pgen.1005041.s023.docx]

**Table S1.** Comparison of rates of fixation based on the two-locus model with genetic drift and the admix’em simulation program at different population sizes.

|  | **The two-locus model with multinomial sampling** | | **admix’em** | |
| --- | --- | --- | --- | --- |
| Diploid population size  (N) | Percent fixing parent 1 genotype ± SE | Average time to fixation  ± SD | Percent fixing parent 1 genotype  ± SE | Average time to fixation  ± SD |
| 1,000 | 51.2 ± 2 | 178 ± 47 | 50.4 ± 2 | 177 ± 44 |
| 10,000 | 50.2 ± 2 | 233 ± 45 | 49.8 ± 2 | 218 ± 30 |

Note – One hybrid incompatibility pair (Figure S2), *s*_1_=*s*_2_=0.1, *f*=0.5, *h*=0.5 for 500 replicate simulations.
