# Supplementary material for: Reproductive Isolation of Hybrid Populations Driven by Genetic Incompatibilities
Source: PLoS Genet. 2015 Mar 13;11(3):e1005041. doi: 10.1371/journal.pgen.1005041 (PMC4359097; doi:10.1371/journal.pgen.1005041)
Supplement: S2 Table — (DOCX) [file pgen.1005041.s024.docx]

**Table S2.** The effect of increasing selection on hybrids on the probability of and time to isolation.

| **Fitness of F1 hybrid** | **Percent isolating ± SE** | **Average time to isolation** ± **SD** |
| --- | --- | --- |
| 0.9 | 47 ± 2 | 203 ± 41 |
| 0.8 | 36 ± 2 | 120 ± 22 |
| 0.7 | 28 ± 2 | 75 ± 16 |
| 0.5 | 12 ± 2 | 49 ± 9 |

Note – Two hybrid incompatibility pairs (Figure S2), *s*_1_=*s*_2,_ N=1000, *f*=0.5, *h*=0.5 for 500

replicate simulations.
