# Supplementary material for: Reproductive Isolation of Hybrid Populations Driven by Genetic Incompatibilities
Source: PLoS Genet. 2015 Mar 13;11(3):e1005041. doi: 10.1371/journal.pgen.1005041 (PMC4359097; doi:10.1371/journal.pgen.1005041)
Supplement: S3 Table — (DOCX) [file pgen.1005041.s025.docx]

**Table S3.** The effect of population size on the probability of

and time to isolation.

| **Diploid population size**  **(N)** | **Percent isolating**  **± SE** | **Average time to isolation**  ± **SD** |
| --- | --- | --- |
| 100 | 39 ± 2 | 121 ± 39 |
| 1000 | 47 ± 2 | 203 ± 41 |
| 10000 | 43 ± 2 | 258 ± 38 |

Note – Two hybrid incompatibility pairs (Figure S2), *s*_1_=*s*_2_=0.1,

*f*=0.5, *h*=0.5 for 500 replicate simulations.
