# Supplementary material for: Reproductive Isolation of Hybrid Populations Driven by Genetic Incompatibilities
Source: PLoS Genet. 2015 Mar 13;11(3):e1005041. doi: 10.1371/journal.pgen.1005041 (PMC4359097; doi:10.1371/journal.pgen.1005041)
Supplement: S4 Table — (DOCX) [file pgen.1005041.s026.docx]

**Table S4.** Effects of asymmetry in selection

on the probability of isolation.

| **Asymmetry of selection** | **Percent isolating**  **± SE** | **Average time to isolation** ± **SD** |
| --- | --- | --- |
| *s*_A_=*s*_B_ | 47 ± 2 | 203 ± 41 |
| *s*_A_=0.5**s*_B_ | 45 ± 2 | 296 ± 67 |
| *s*_A_=0.4**s*_B_ | 42 ± 2 | 332 ± 88 |
| *s*_A_=0.3**s*_B_ | 43 ± 2 | 420 ± 99 |
| *s*_A_=0.2**s*_B_ | 36 ± 2 | 543 ± 106 |
| *s*_A_=0**s*_B_ | 0.6 ± 0.3 | 1380 ± 360 |

Note – Two hybrid incompatibility pairs

(Figure S1), *s*_A_=0.1, N=1000, *f*=0.5, *h*=0.5

for 500 replicate simulations.
