# Supplementary material for: Reproductive Isolation of Hybrid Populations Driven by Genetic Incompatibilities
Source: PLoS Genet. 2015 Mar 13;11(3):e1005041. doi: 10.1371/journal.pgen.1005041 (PMC4359097; doi:10.1371/journal.pgen.1005041)
Supplement: S5 Table — (DOCX) [file pgen.1005041.s027.docx]

**Table S5**. The effect of variation in dominance.

| **Dominance** | **Percent isolating**  **± SE** | **Average time to isolation** ± **SD** |
| --- | --- | --- |
| *h* = 0.5 | 47 ± 2 | 203 ± 41 |
| *h* = Uniform(0,1) | 48 ± 2 | 244 ± 60 |
| *h* ∈ (0, 0.5, 1) | 42 ± 2 | 291 ± 69 |

Note – Two hybrid incompatibility pairs (Figure S2),

*s*_1_=*s*_2_=0.1, N=1000, *f*=0.5, for 500 replicate simulations.
