# Supplementary material for: Reproductive Isolation of Hybrid Populations Driven by Genetic Incompatibilities
Source: PLoS Genet. 2015 Mar 13;11(3):e1005041. doi: 10.1371/journal.pgen.1005041 (PMC4359097; doi:10.1371/journal.pgen.1005041)
Supplement: S6 Table — (DOCX) [file pgen.1005041.s028.docx]

**Table S6**. Effect of linkage between hybrid incompatibilities on

the probability of and time to isolation.

| **Linkage scenario**  **(Figure S10)** | **Genetic**  **distance** | **Percent isolating ± SE** | **Average time to isolation** ± **SD** |
| --- | --- | --- | --- |
| Scenario 1 | 50 cM | 46 ± 2 | 195 ± 44 |
| Scenario 1 | 10 cM | 43 ± 2 | 213 ± 46 |
| Scenario 1 | 1 cM | 45 ± 2 | 198 ± 49 |
| Scenario 2 | 50 cM | 42 ± 2 | 206 ± 48 |
| Scenario 2 | 10 cM | 32 ± 2 | 212 ± 47 |
| Scenario 2 | 1 cM | 8 ± 1 | 207 ± 49 |

Note – A visual representation of the linkage scenarios can be found

in Figure S10. Simulation parameters: Two hybrid incompatibility pairs

(Figure S2), *s*_1_=*s*_2_=0.1. N=1000, *f*=0.5, *h*=0.5 for 500 replicate simulations.
