# Supplementary material for: Reproductive Isolation of Hybrid Populations Driven by Genetic Incompatibilities
Source: PLoS Genet. 2015 Mar 13;11(3):e1005041. doi: 10.1371/journal.pgen.1005041 (PMC4359097; doi:10.1371/journal.pgen.1005041)
Supplement: S7 Table — (DOCX) [file pgen.1005041.s029.docx]

**Table S7.** The effect of on-going and bursts of parental immigration on the probability of

and time to isolation.

| **Migration scenario** | **Hybrid zone structure** | **Percent isolating ± SE** | **Average time to isolation** ± **SD** |
| --- | --- | --- | --- |
| Continuous (4*Nm*_1,2_=8) | Figure S3 | 38 ± 2 | 255 ± 65 |
| Continuous (4*Nm*_1,2_=12) | Figure S3 | 37 ± 2 | 393 ± 97 |
| Continuous (4*Nm*_1,2_*=*20) | Figure S3 | 4 ± 1 | 589 ± 81 |
| Burst – generation 25 (4*Nm*=400) | Figure S3 | 38 ± 2 | 205 ± 35 |
| Burst – generation 50 (4*Nm*=400) | Figure S3 | 37 ± 2 | 213 ± 53 |
| Burst – generation 75 (4*Nm*=400) | Figure S3 | 38 ± 2 | 195 ± 44 |
| Continuous (4*Nm*_1,2_=8) | Figure S13 | 43 ± 2 | 262 ± 82 |
| Continuous (4*Nm*_1,2_=12) | Figure S13 | 37 ± 2 | 281 ± 91 |
| Continuous (4*Nm*_1,2_*=*20) | Figure S13 | 3 ± 1 | 533 ± 132 |

Note – Two hybrid incompatibility pairs (Figure S2), *s*_1_=*s*_2_=0.1, N=1000, *f*=0.5, *h*=0.5

for 500 replicate simulations. For the “Burst” models, all migration occurs in one generation.
