# Supplementary material for: Reproductive Isolation of Hybrid Populations Driven by Genetic Incompatibilities
Source: PLoS Genet. 2015 Mar 13;11(3):e1005041. doi: 10.1371/journal.pgen.1005041 (PMC4359097; doi:10.1371/journal.pgen.1005041)
Supplement: S8 Table — (DOCX) [file pgen.1005041.s030.docx]

**Table S8.** Independently formed hybrid populations can evolve reproductive

isolation from each other.

| **Number of incompatibility pairs** | **Migration rate** | **Percent reciprocally isolated ± SE** |
| --- | --- | --- |
| 2 | 4*Nm*=0 | 50 ± 5 |
| 2 | 4*Nm*=8 | 24 ± 4 |
| 2 | 4*Nm*=12 | 10 ± 3 |
| 3 | 4*Nm*=0 | 81 ± 4 |
| 3 | 4*Nm*=8 | 72 ± 4 |
| 3 | 4*Nm*=12 | 46 ± 5 |

Note – Two or three hybrid incompatibility pairs (Figure S2),

*s*_1_=*s*_2_=0.1, N=1000, *f*=0.5. Simulations were conducted until

100 replicates were generated in which both hybrid

populations were isolated from parentals.
