# Supplementary material for: Reproductive Isolation of Hybrid Populations Driven by Genetic Incompatibilities
Source: PLoS Genet. 2015 Mar 13;11(3):e1005041. doi: 10.1371/journal.pgen.1005041 (PMC4359097; doi:10.1371/journal.pgen.1005041)
Supplement: S9 Table — (DOCX) [file pgen.1005041.s031.docx]

**Table S9.** Abundance of hybrids in several previously studied natural hybrid populations.

| **Parent species** | **Hybrid abundance** | **References** |
| --- | --- | --- |
| *Alosa pseudoharengas – A. aestivalis* | 100% | [1] |
| *Cyprinella lutrensis – C. venusta* | 84% | [2] |
| *Geum rivale – G. urbanum* | 47% | [3] |
| *Odocoileus hemionus* (subspecies mule and black-tail deer) | 44% | [4] |
| *Oncorhynchus mykiss – O. clarki clarki* | 23% | [5] |
| *Oncorhynchus mykiss – O. clarki lewisi* | 3-57% | [6] |
| *Picea sitchensis – P. glauca* | 88% | [7] |
| *Senecio aethnensis – S. chrysanthemifolius* | 100% | [8] |
| *Quercus grisea – Q. gambelli* | 89% | [9] |
| *Xiphophorus birchmanni* – *X. malinche* | 55-100% | [10] |

**Table S9 References**

1. Hasselman DJ, Argo EE, McBride MC, Bentzen P, Schultz TF, et al. (2014) Human disturbance causes the formation of a hybrid swarm between two naturally sympatric fish species. Mol Ecol 23: 1137–1152.

2. Ward JL, Blum MJ, Walters DM, Porter BA, Burkhead N, et al. (2012) Discordant introgression in a rapidly expanding hybrid swarm. Evolutionary Applications 5: 380-392.

3. Ruhsam M, Hollingsworth PM, Ennos RA (2011) Early evolution in a hybrid swarm between outcrossing and selfing lineages in Geum. Heredity 107: 246-255.

4. Latch EK, Kierepka EM, Heffelfinger JR, Rhodes OE (2011) Hybrid swarm between divergent lineages of mule deer (Odocoileus hemionus). Molecular Ecology 20: 5265-5279.

5. Moore ME, Goetz FA, Van Doornik DM, Tezak EP, Quinn TP, et al. (2010) Early Marine Migration Patterns of Wild Coastal Cutthroat Trout (Oncorhynchus clarki clarki), Steelhead Trout (Oncorhynchus mykiss), and Their Hybrids. Plos One 5.

6. Rubidge EM, Taylor EB (2004) Hybrid zone structure and the potential role of selection in hybridizing populations of native westslope cutthroat trout (Oncorhynchus clarki lewisi) and introduced rainbow trout (O-mykiss). Molecular Ecology 13: 3735-3749.

7. Hamilton JA, Lexer C, Aitken SN (2013) Genomic and phenotypic architecture of a spruce hybrid zone (Picea sitchensis P. glauca). Molecular Ecology 22: 827-841.

8. James JK, Abbott RJ (2005) Recent, allopatric, homoploid hybrid speciation: The origin of Senecio squalidus (Asteraceae) in the British Isles from a hybrid zone on Mount Etna, Sicily. Evolution 59: 2533-2547.

9. Howard DJ, Preszler RW, Williams J, Fenchel S, Boecklen WJ (1997) How discrete are oak species? Insights from a hybrid zone between Quercus grisea and Quercus gambelii. Evolution 51: 747-755.

10. Schumer M, Cui R, Powell D, Dresner R, Rosenthal G, et al. (2014) High-resolution Mapping Reveals Hundreds of Genetic Incompatibilities in Hybridizing Fish Species. eLife 3.
