# Supplementary material for: Reproductive Isolation of Hybrid Populations Driven by Genetic Incompatibilities
Source: PLoS Genet. 2015 Mar 13;11(3):e1005041. doi: 10.1371/journal.pgen.1005041 (PMC4359097; doi:10.1371/journal.pgen.1005041)
Supplement: S10 Table — (DOCX) [file pgen.1005041.s032.docx]

**Table S10.** Parental preferences for conspecifics reduce the frequency of

hybrid reproductive isolation.

| **Parental preference** | **Percent isolating ± SE** | **Average time to isolation** ± **SD** |
| --- | --- | --- |
| None | 47± 2% | 203 ± 41 |
| 25 attempts | 40 ± 2% | 264 ± 67 |
| 50 attempts | 29 ± 2% | 250 ± 68 |
| 75 attempts | 24 ± 2% | 269 ± 55 |

Note – Two hybrid incompatibility pairs (Figure S2), 4*Nm_1_*=4*Nm_2_*=8,

*s*_1_=*s*_2_=0.1, N=1000, *f*=0.5, *h*=0.5 for 500 replicate simulations.
