# Supplementary material for: Reproductive Isolation of Hybrid Populations Driven by Genetic Incompatibilities
Source: PLoS Genet. 2015 Mar 13;11(3):e1005041. doi: 10.1371/journal.pgen.1005041 (PMC4359097; doi:10.1371/journal.pgen.1005041)
Supplement: S11 Table — (DOCX) [file pgen.1005041.s033.docx]

**Table S11.** The effect of increasing selection on hybrids on the probability of and time to isolation under the underdominant inversion model

| **Fitness of F1 hybrid** | **Percent isolating ± SE** | **Average time to isolation** ± **SD** |
| --- | --- | --- |
| 0.9 | 43 ± 2 | 212 ± 59 |
| 0.8 | 37 ± 2 | 118 ± 25 |
| 0.7 | 33 ± 2 | 76 ± 13 |
| 0.5 | 17 ± 2 | 48 ± 10 |

Note – Two underdominant inversions, *s*_1_=*s*_2,_ N=1000, *f*=0.5, for 500

replicate simulations.
